# Supplementary material for: Diketopiperazine and Diphenylether Derivatives from Marine Algae-Derived Aspergillus versicolor OUCMDZ-2738 by Epigenetic Activation
Source: Mar Drugs. 2018 Dec 22;17(1):6. doi: 10.3390/md17010006 (PMC6356248; doi:10.3390/md17010006)
Supplement: Supplementary file 1 [file marinedrugs-17-00006-s001.pdf]

Supplementary Materials

Diketopiperazine and Diphenylether Derivatives from Marine Algae-Derived *Aspergillus versicolor* OUCMDZ-2738 by Epigenetic Activation

Wen Liu [1,2,†], Liping Wang [2,†], Bin Wang [1], Yanchao Xu [2], Guoliang Zhu [3,4], Mengmeng Lan [3], Weiming Zhu [2,3,5,*] and Kunlai Sun [1,*]

1 Zhejiang Provincial Engineering Technology Research Center of Marine Biomedical Products, School of Food and Pharmacy, School of Marine Science and Technology, Zhejiang Ocean University, Zhoushan 316022, China; yxlliuwen@outlook.com (W.L.); wangbin4159@hotmail.com (B.W.)

2 State Key Laboratory of Functions and Applications of Medicinal Plants, Guizhou Medical University, Guiyang 550014, China; lipingw2006@163.com (L.W.); m18586818694@163.com (Y.X.)

3 Key Laboratory of Marine Drugs, Ministry of Education of China, School of Medicine and Pharmacy, Ocean University of China, Qingdao 266003, China; zhuguoliang@ecust.edu.cn (G.Z.); lanmengmeng90@163.com (M.L.)

4 State Key Laboratory of Bioreactor Engineering, East China University of Science and Technology, Shanghai 200237, China

5 Open Studio for Druggability Research of Marine Natural Products, Pilot National Laboratory for Marine Science and Technology (Qingdao), Qingdao, 266237, China

† These authors contributed equally to this paper.

* Correspondence: weimingzhu@ouc.edu.cn (W.Z.); sunqinlai@126.com (K.S.); Tel./Fax: +86-532-8203-1268 (W.Z.); +86-580-255-4781 (K.S.)

List of Supplementary Materials

Figure S1. HRESIMS spectrum of compound **1** S3
Figure S2. ^{1}H NMR spectrum of compound **1** in DMSO-d_6........S3
Figure S3. ^{13}C NMR spectrum of compound **1** in DMSO-d_6 ....S4
Figure S4. DEPT spectrum of compound **1** in DMSO-d_6........S4
Figure S5. HSQC spectrum of compound **1** in DMSO-d_6 ....S5
Figure S6. ^{1}H–^{1}H COSY spectrum of compound **1** in DMSO-d_6........S5
Figure S7. HMBC spectrum of compound **1** in DMSO-d_6 ....S6
Figure S8. HRESIMS spectrum of compound **2**........S6
Figure S9. ^{1}H NMR spectrum of compound **2** in methanol-d_4 ....S7
Figure S10. ^{13}C NMR spectrum of compound **2** in methanol-d_4 ....S7
Figure S11. DEPT spectrum of compound **2** in methanol-d_4........S8
Figure S12. HSQC spectrum of compound **2** in methanol-d_4........S8
Figure S13. ^{1}H–^{1}H COSY spectrum of compound **2** in methanol-d_4 ....S9
Figure S14. HMBC spectrum of compound **2** in methanol-d_4........S9
Figure S15. The chiral HPLC analysis of compounds **2–5**........S10
Figure S16. The chiral HPLC analysis of compounds **7–9**........S12
Figure S17. DFT-optimized structures for low-energy conformers of compounds **2–5**......S12
Figure S18. Phylogenetic tree mapping for the *Aspergillus versicolor* OUCMDZ-2738....S14
Figure S19. ^{1}H NMR spectrum of compound **12** in DMSO-d_6........S14
Figure S20. ^{13}C NMR spectrum of compound **12** in DMSO-d_6 ....S15
Figure S21. HSQC spectrum of compound **12** in DMSO-d_6 ....S15
Figure S22. HMBC spectrum of compound **12** in DMSO-d_6 ....S16
Figure S23. ROESY spectrum of compound **12** in DMSO-d_6........S16
Figure S24. HRESIMS spectrum of compound **12a**........S17
Figure S25. ^{1}H NMR spectrum of compound **12a** in DMSO-d_6........S17
Figure S26. ^{13}C NMR spectrum of compound **12a** in DMSO-d_6 ....S18
Figure S27. HSQC spectrum of compound **12a** in DMSO-d_6 ....S18
Figure S28. ROESY spectrum of compound **12a** in DMSO-d_6........S19
Figure S29. HMBC spectrum of compound **12a** in DMSO-d_6 ....S19
Figure S30. The UPLC–MS Analysis of Fermented Extracts........S20
Figure S31. Retention times of compounds **1–12** in the same elution gradient with original extract fingerprint.. ....S20
The physical properties of the known compounds (3–11):........S22

Figure S1. HRESIMS spectrum of compound **1**.

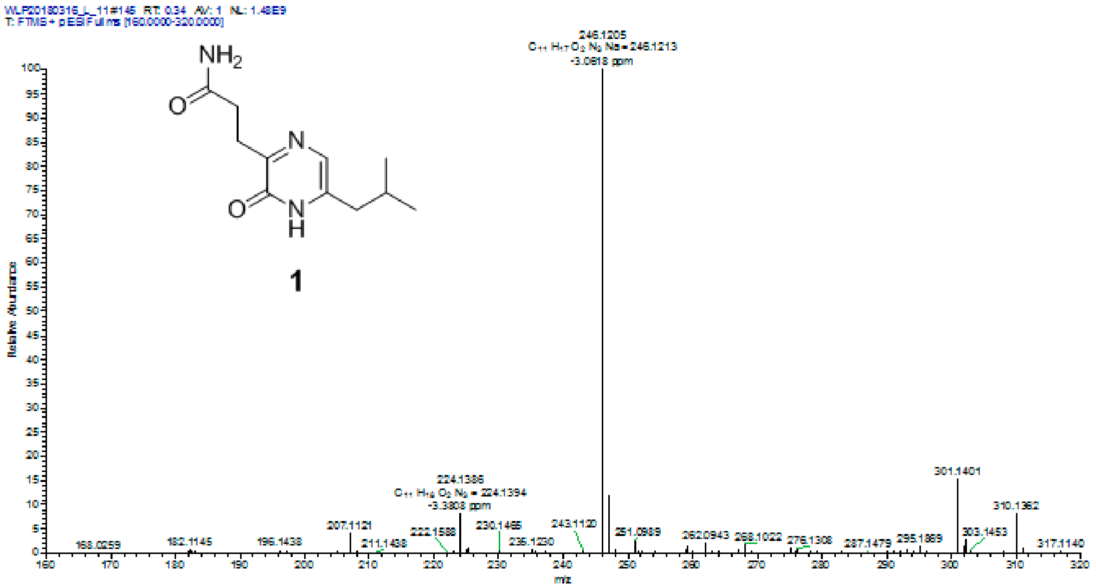

Figure S2. ^{1}H NMR spectrum of compound **1** in DMSO-d_6.

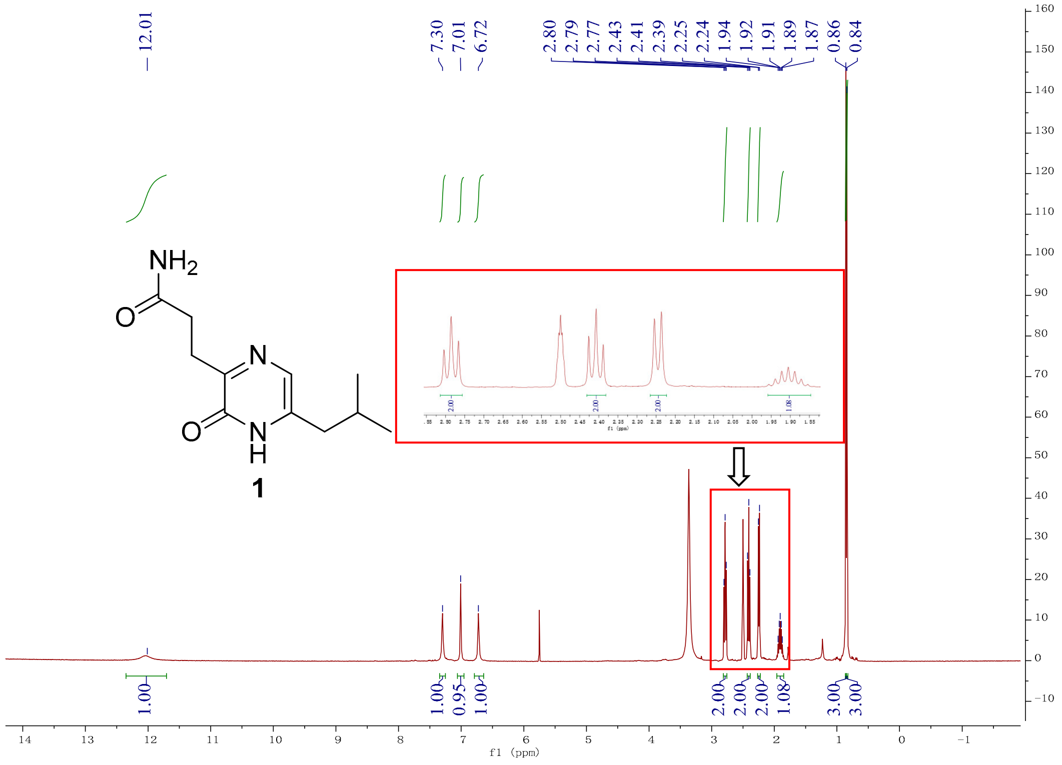

Figure S3. ^{13}C NMR spectrum of compound **1** in DMSO-d_6.

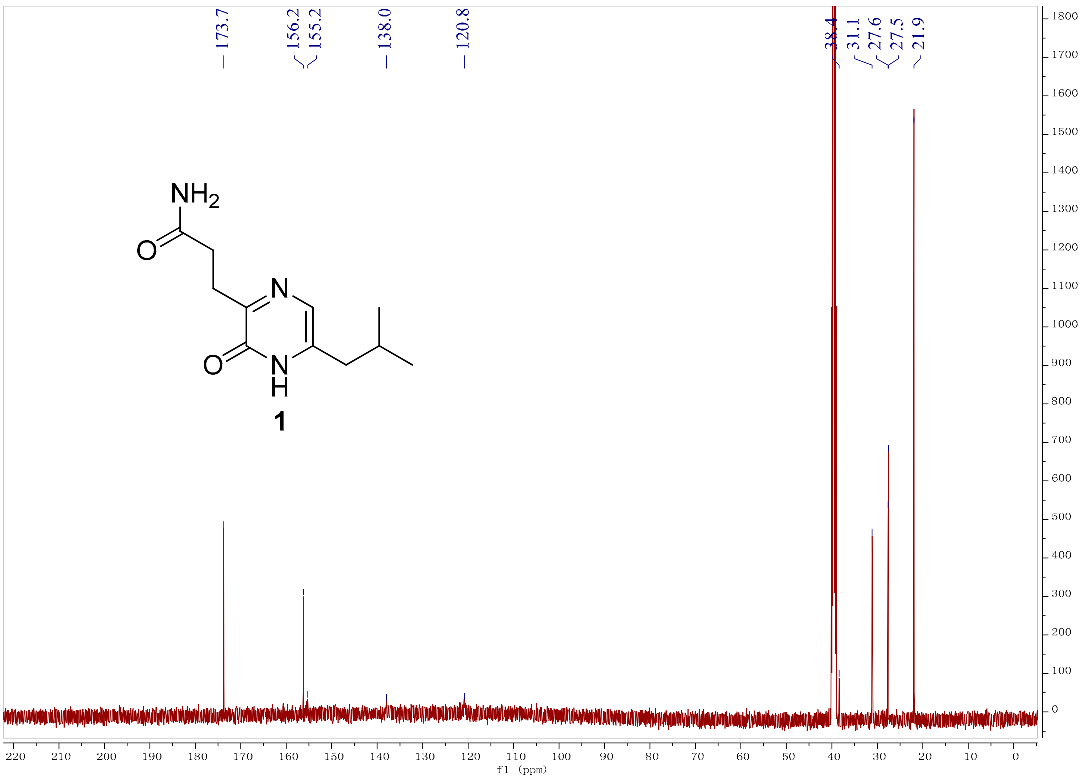

Figure S4. DEPT spectrum of compound **1** in DMSO-d_6.

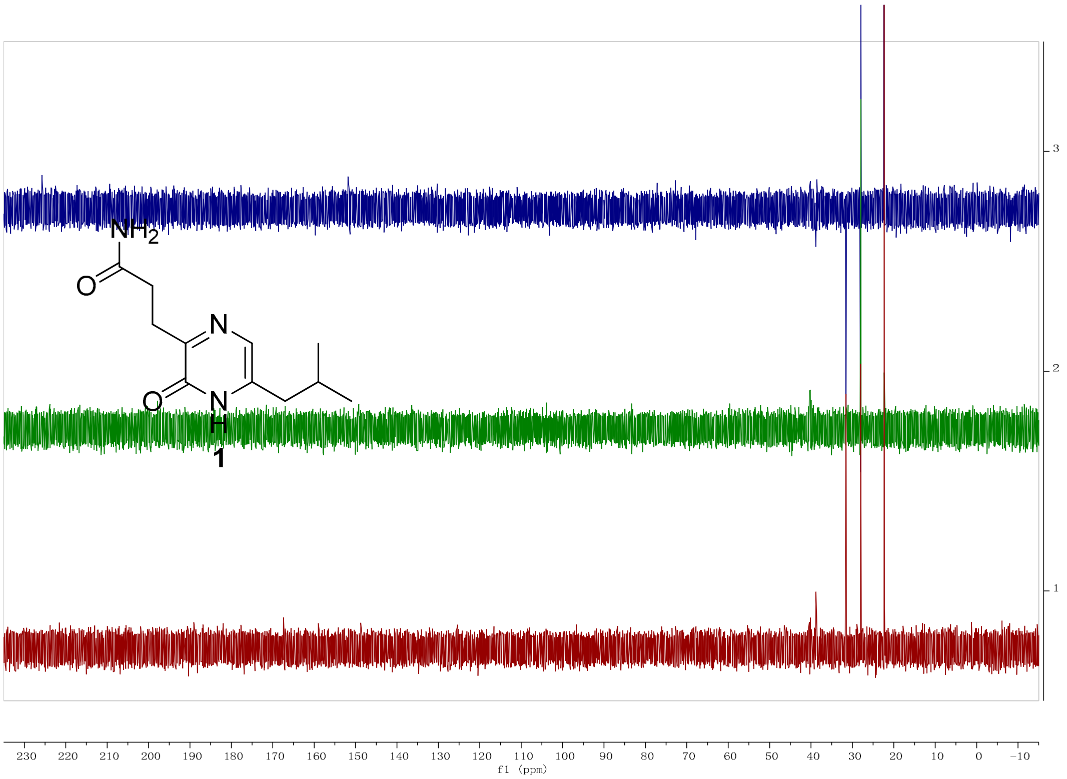

Figure S5. HSQC spectrum of compound **1** in DMSO-d_6.

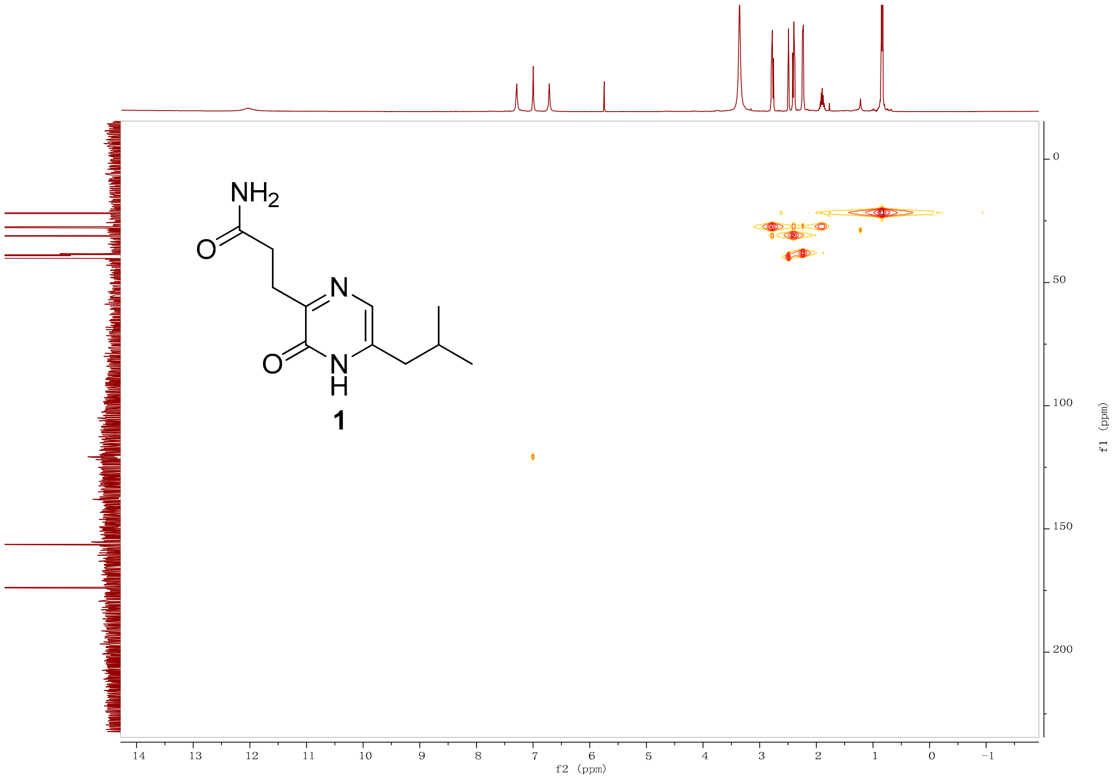

Figure S6. ^{1}H–^{1}H COSY spectrum of compound **1** in DMSO-d_6.

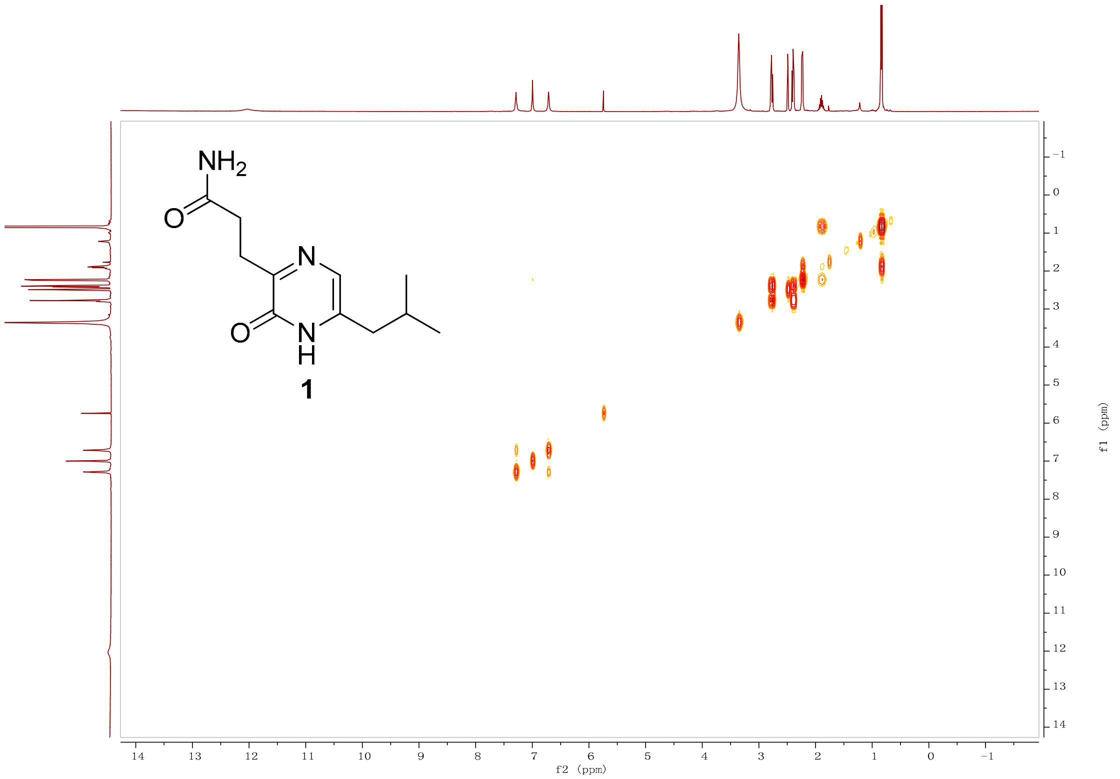

Figure S7. HMBC spectrum of compound **1** in DMSO-d_6.

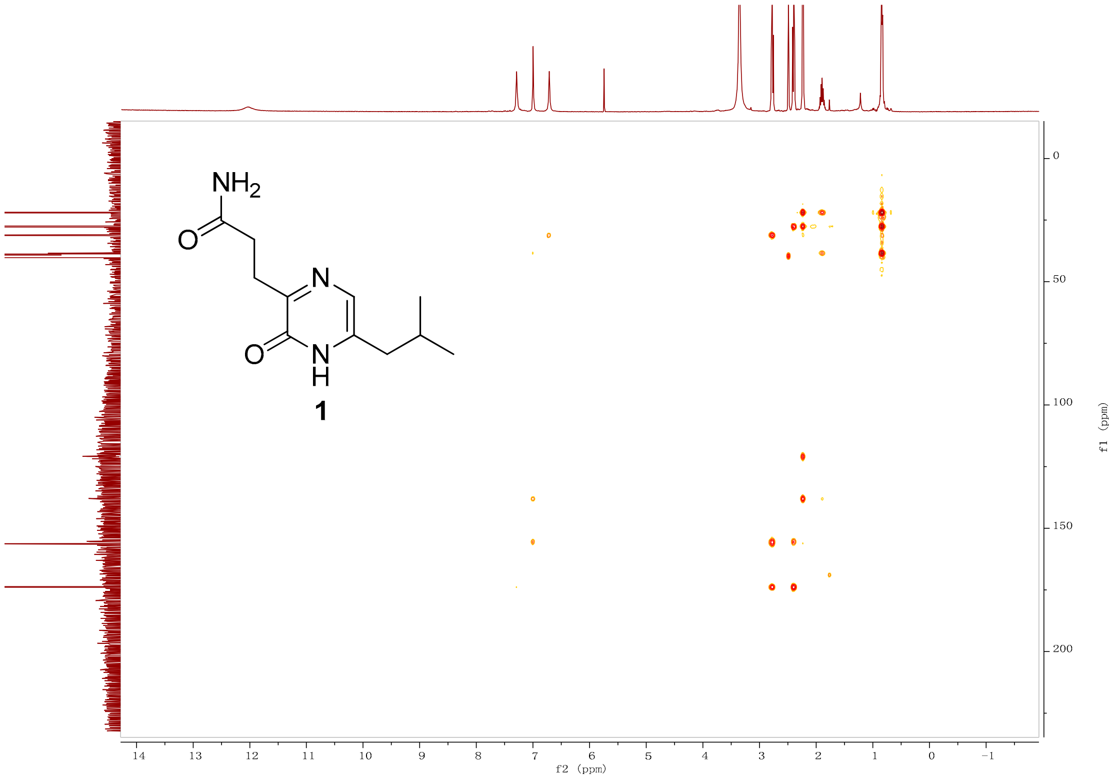

Figure S8. HRESIMS spectrum of compound **2**.

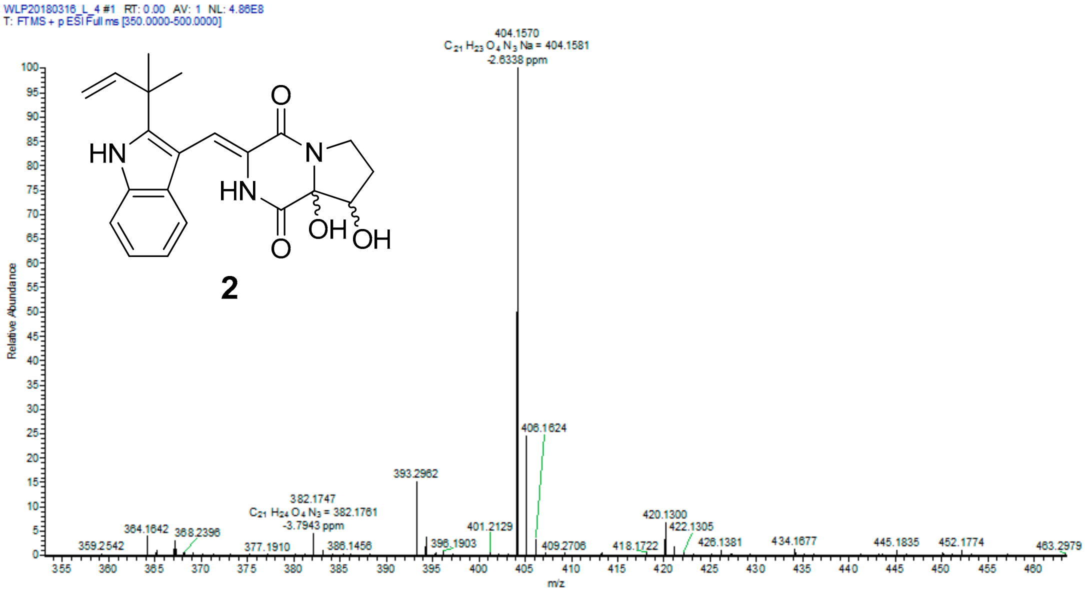

Figure S9. ^{1}H NMR spectrum of compound **2** in methanol-d_4.

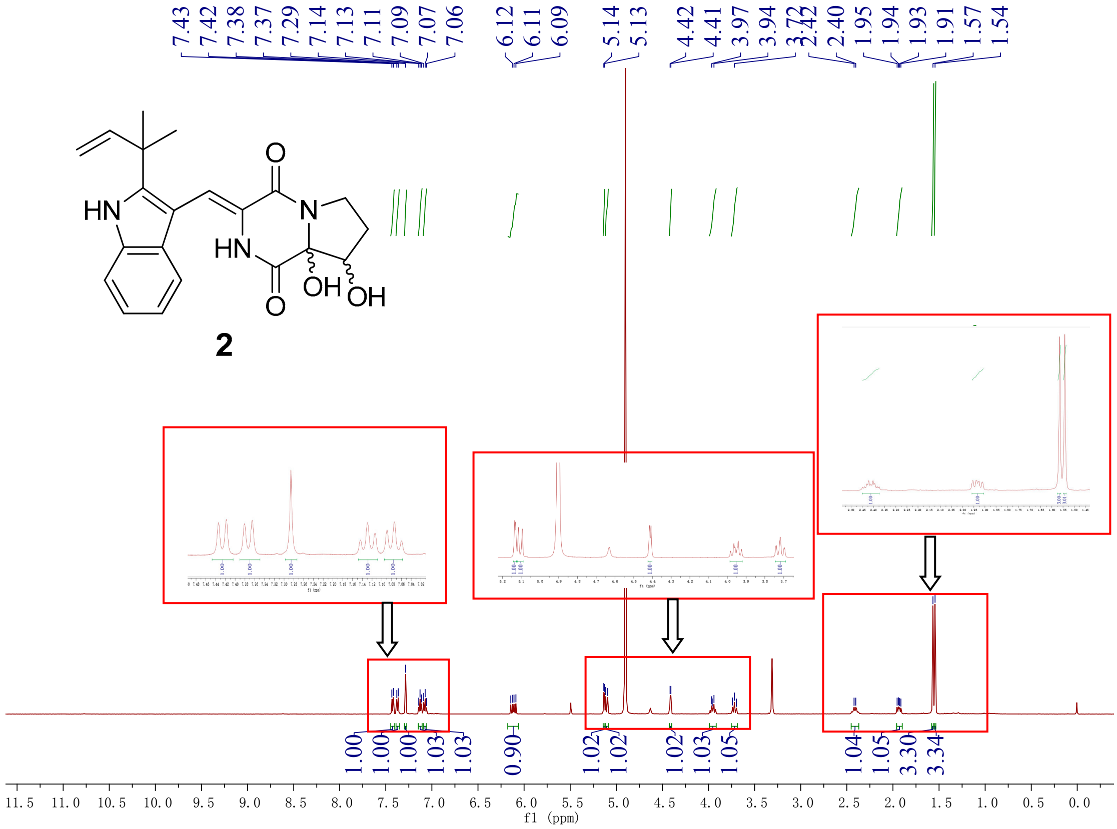

Figure S10. ^{13}C NMR spectrum of compound **2** in methanol-d_4.

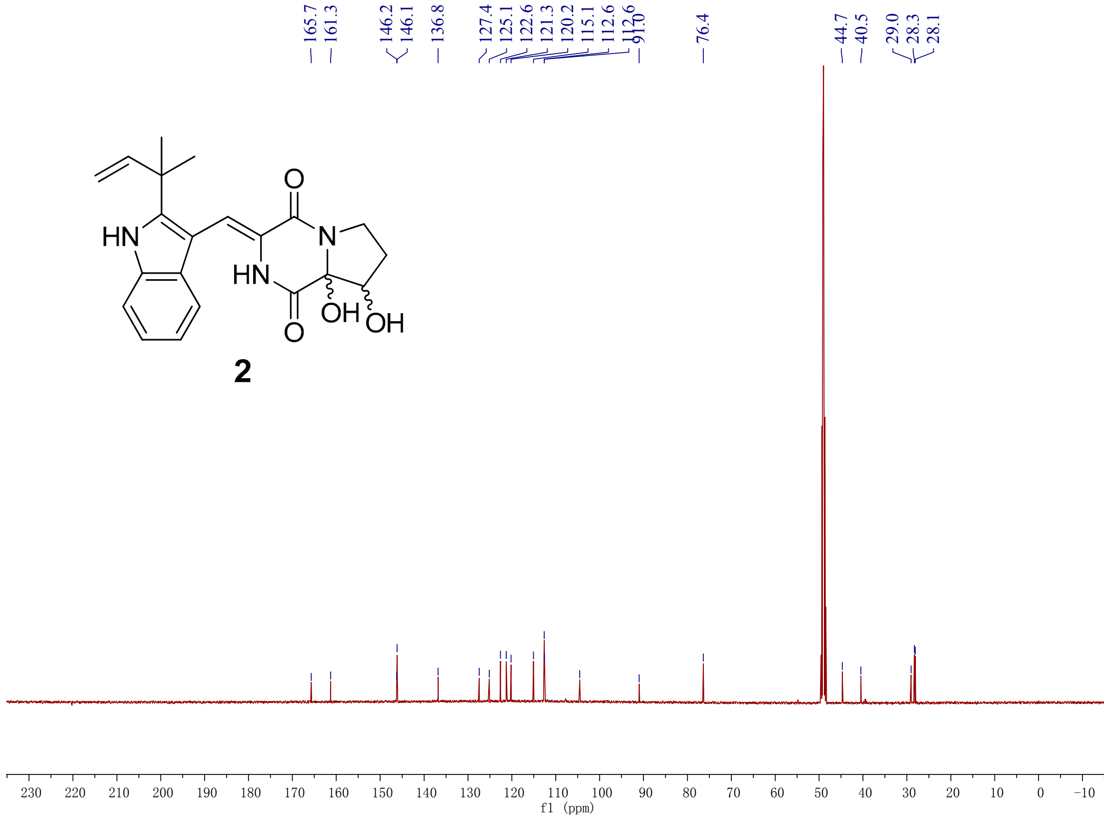

Figure S11. DEPT spectrum of compound **2** in methanol-d_4.

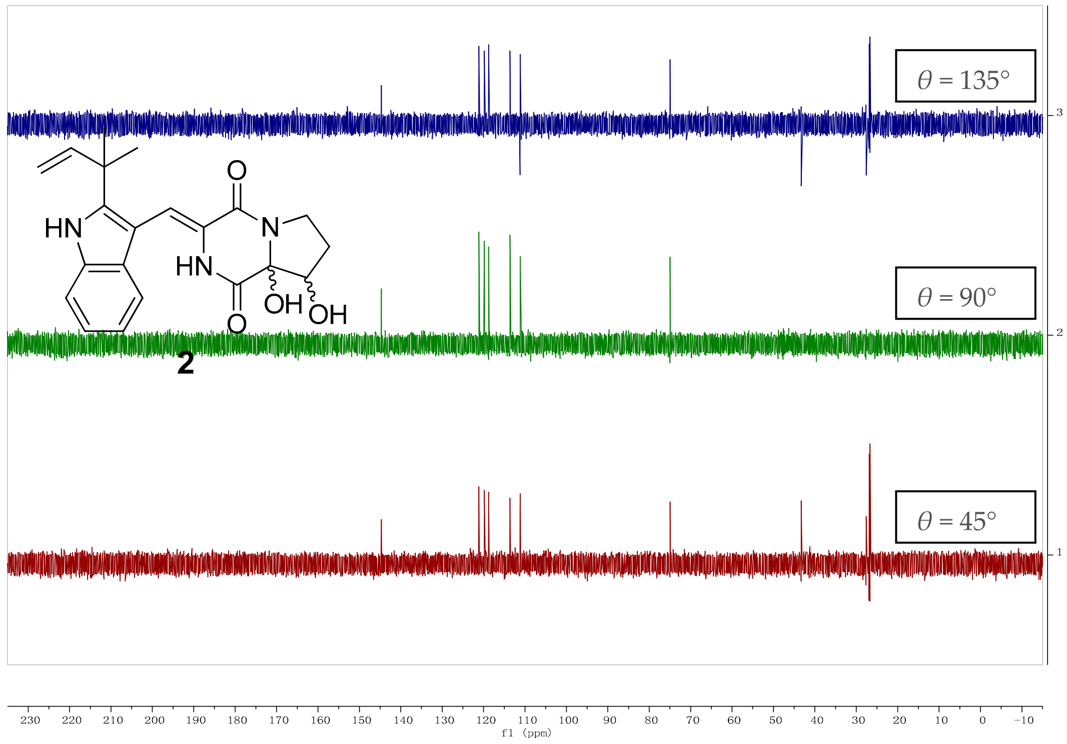

Figure S12. HSQC spectrum of compound **2** in methanol-d_4.

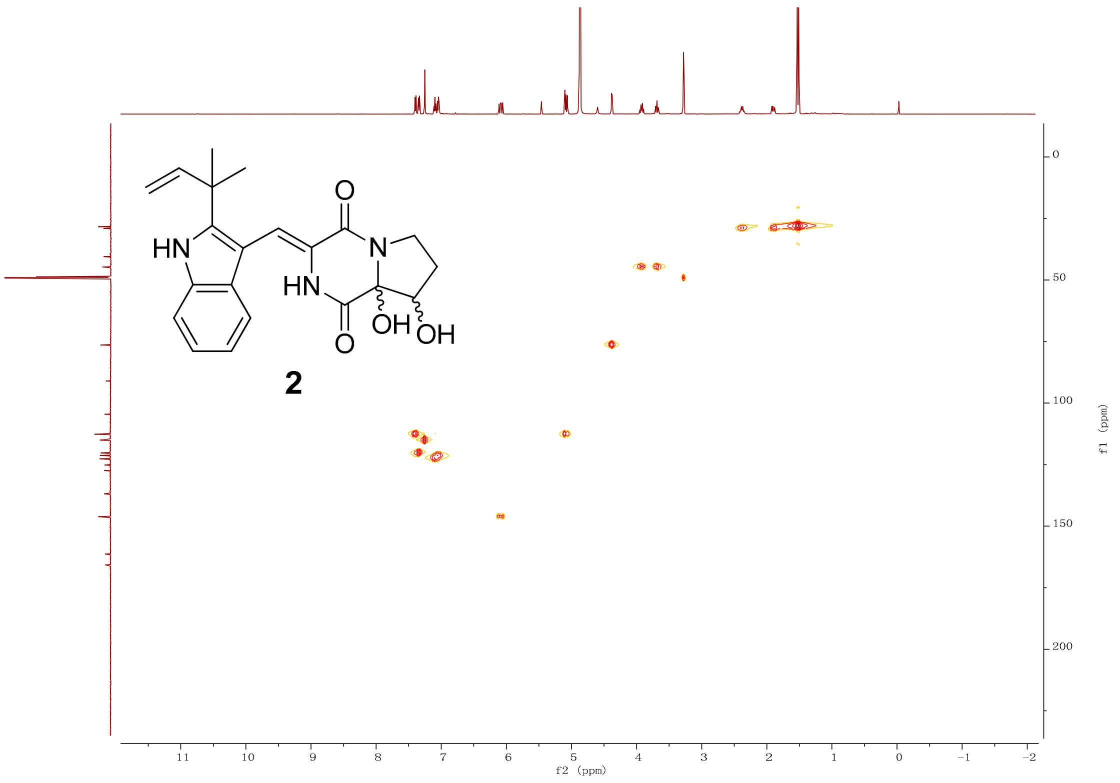

Figure S13. ^{1}H–^{1}H COSY spectrum of compound **2** in methanol-d_4.

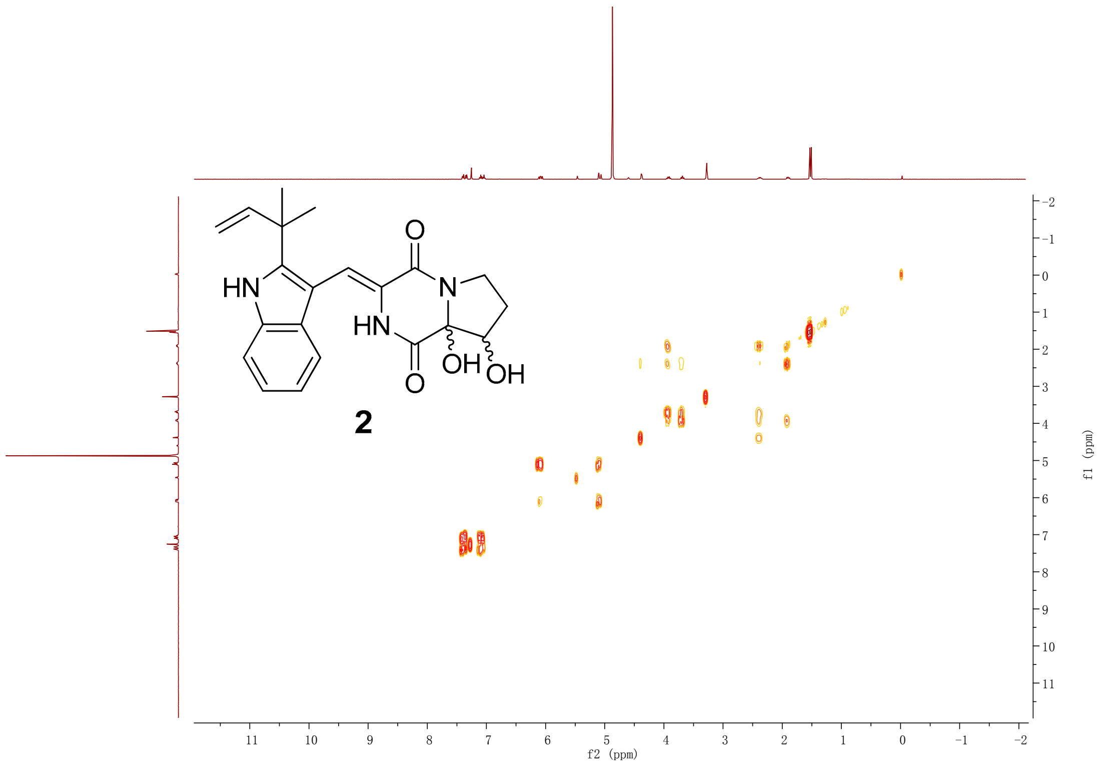

Figure S14. HMBC spectrum of compound **2** in methanol-d_4.

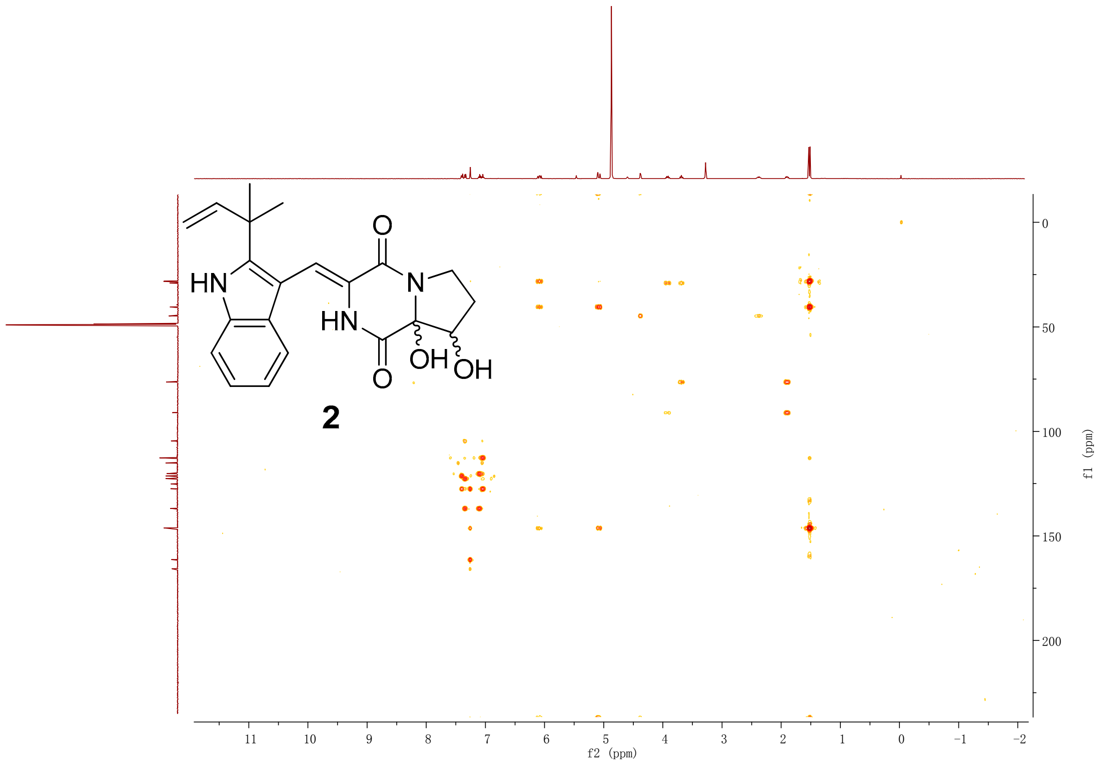

Figure S15. The chiral HPLC analysis of compounds **2–5**.

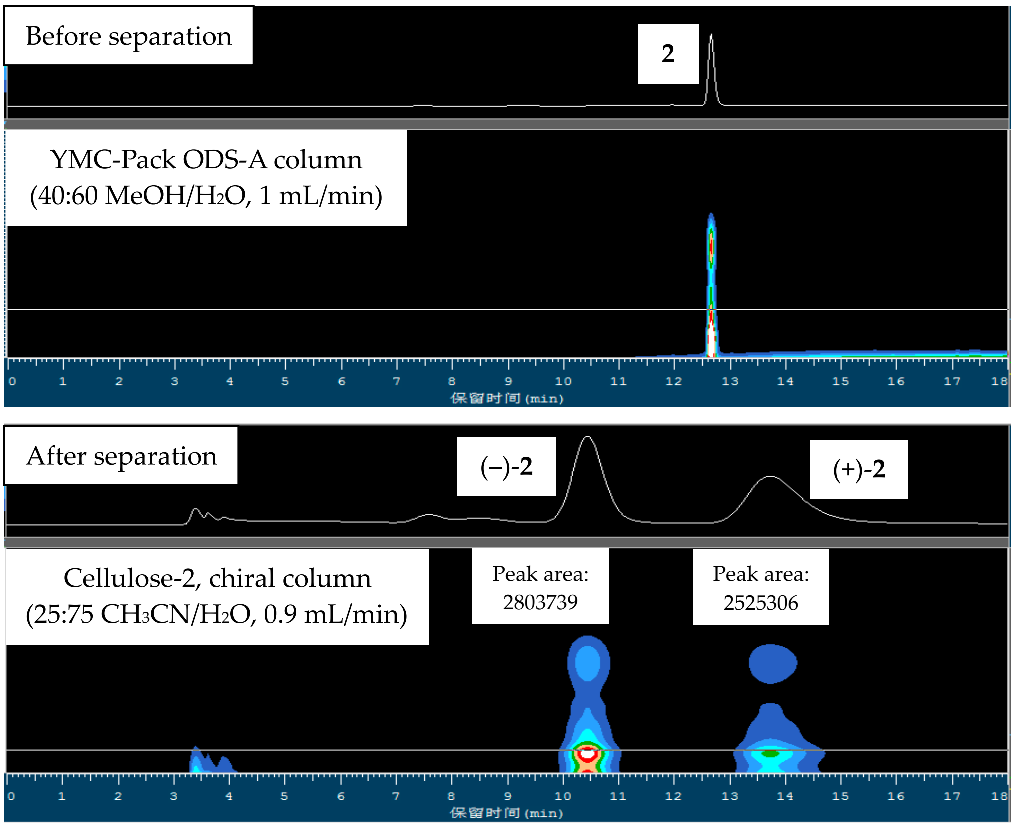

Figure S15A. The chiral HPLC analysis of **2**.

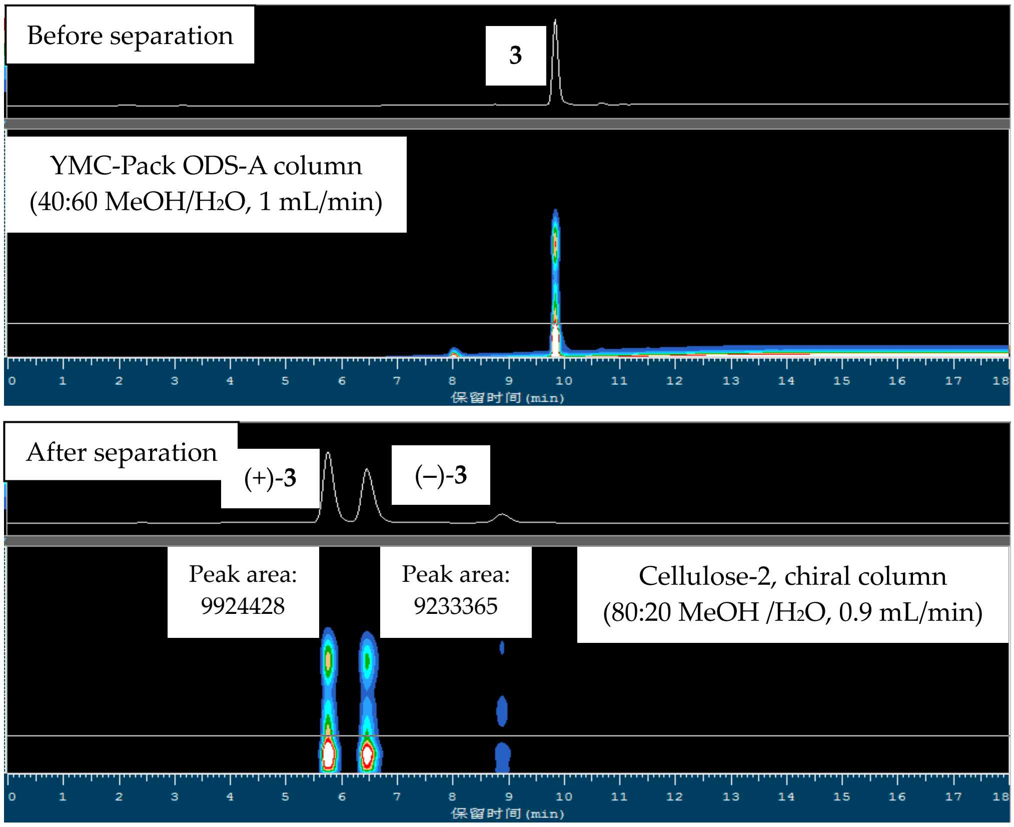

Figure S15B. The chiral HPLC analysis of **3**.

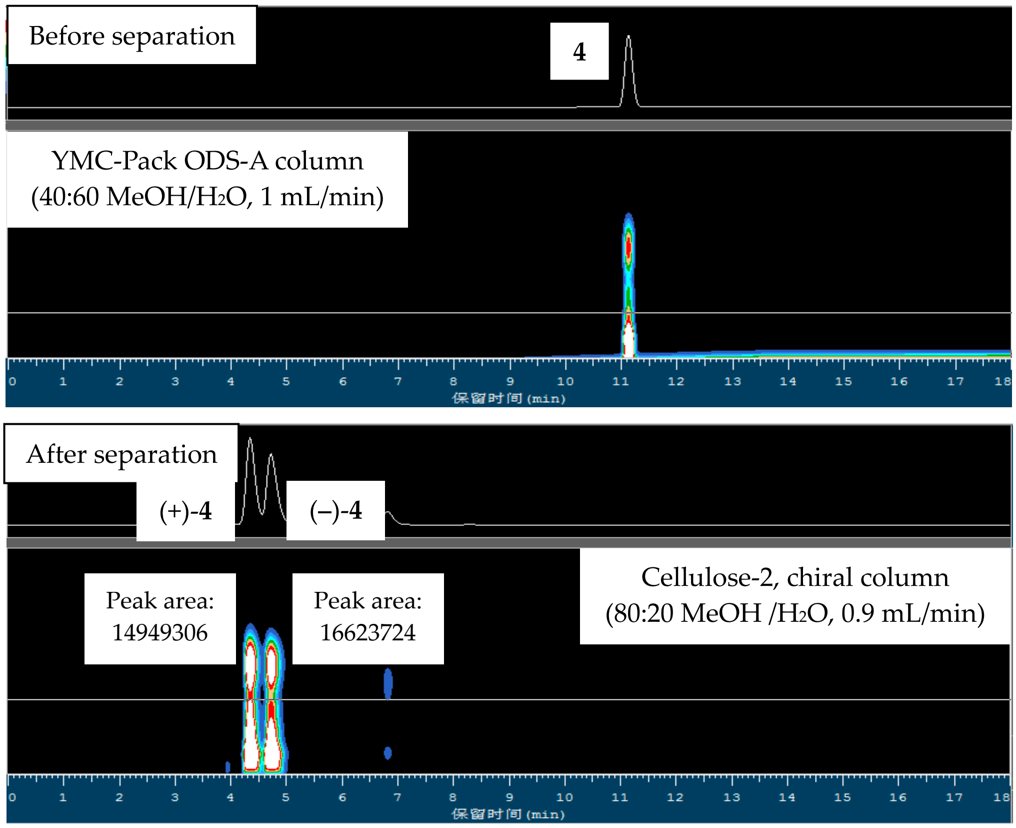

Figure S15C. The chiral HPLC analysis of **4**.

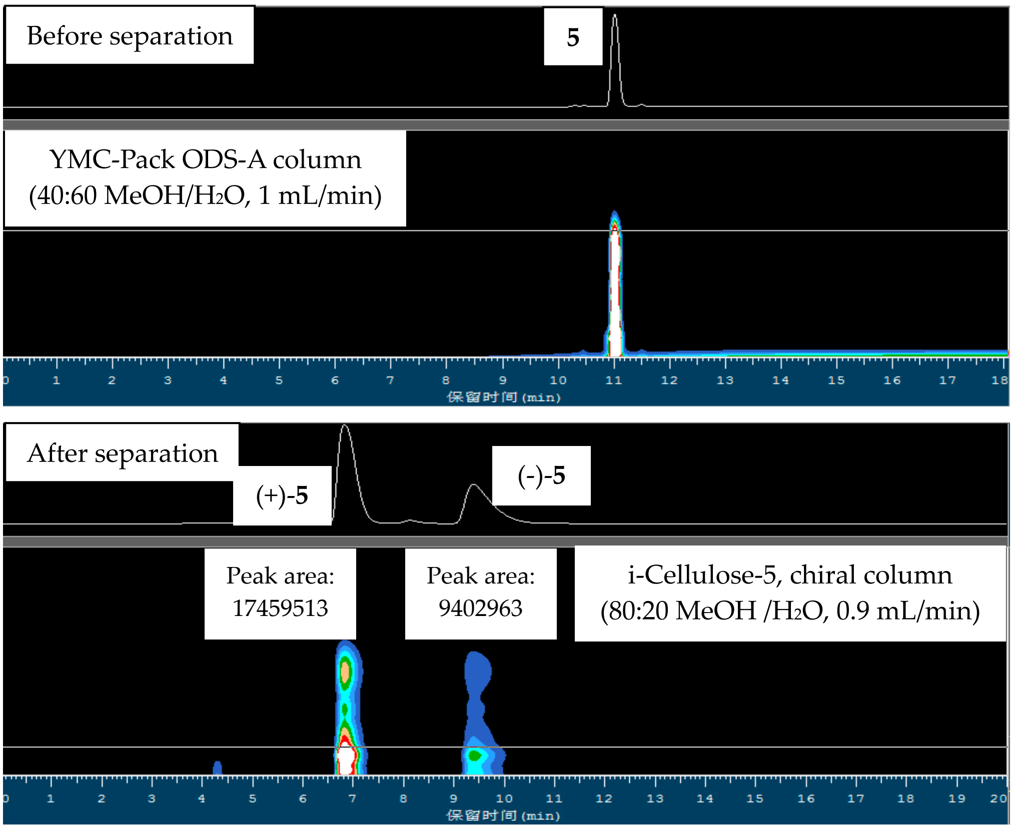

Figure S15D. The chiral HPLC analysis of **5**.

Figure S16. The chiral HPLC analysis of compounds **7–9**.

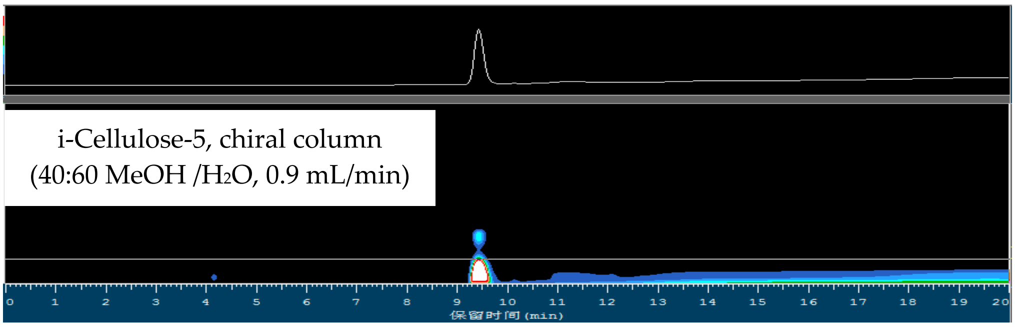

Figure S16A. The chiral HPLC analysis of **7**.

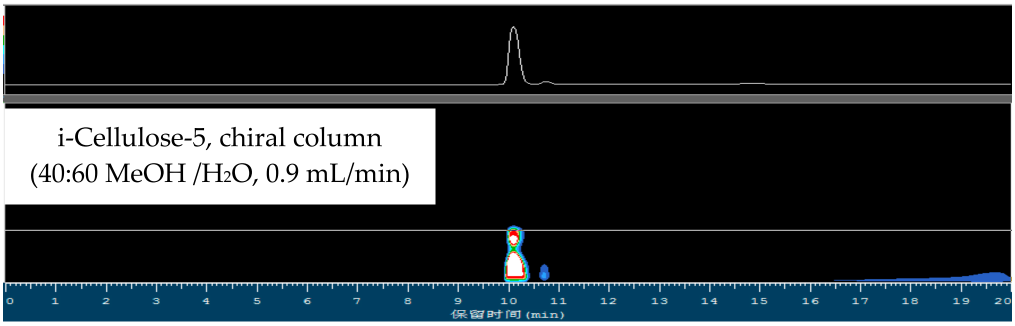

Figure S16B. The chiral HPLC analysis of **8**

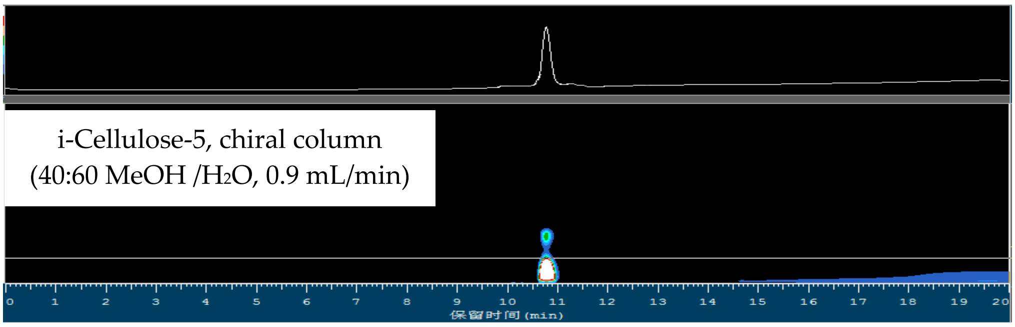

Figure S16C. The chiral HPLC analysis of **9**.

Figure S17. DFT-optimized structures for low-energy conformers of compounds **2–5**.

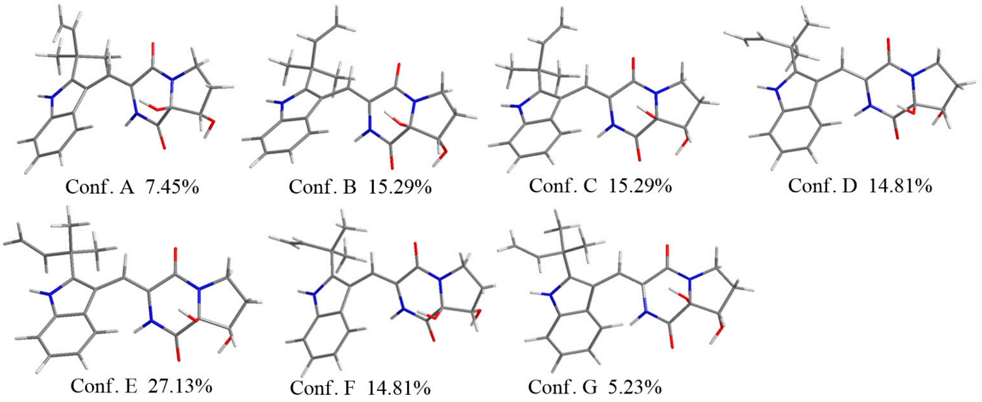

Figure S17A. DFT-optimized structures for low-energy conformers of compound **2** at B3LYP/6-31G(d) level in methanol (PCM).

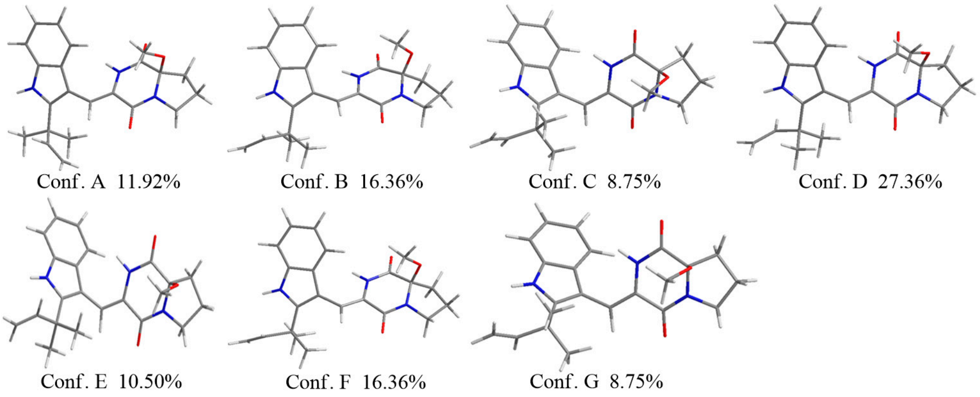

Figure S17B. DFT-optimized structures for low-energy conformers of compound **3** at B3LYP/6-31G(d) level in methanol (PCM).

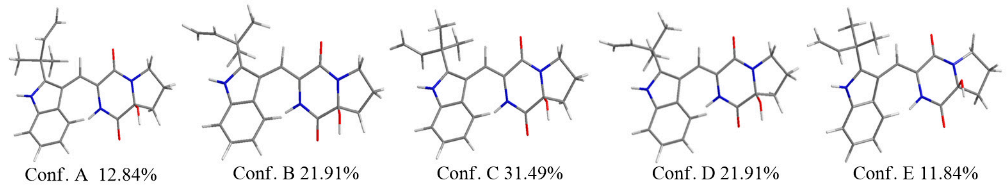

Figure S17C. DFT-optimized structures for low-energy conformers of compound **4** at B3LYP/6-31G(d) level in methanol (PCM).

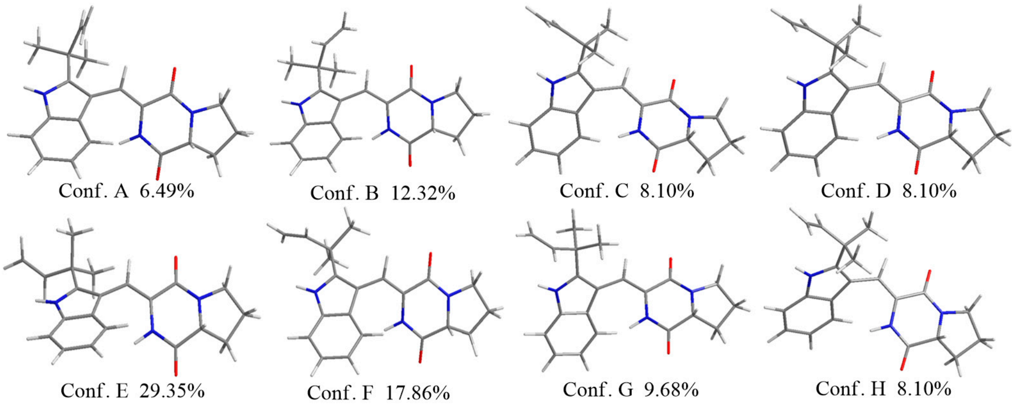

Figure S17D. DFT-optimized structures for low-energy conformers of compound **5** at B3LYP/6-31G(d) level in methanol (PCM).

Figure S18. Phylogenetic tree mapping for the *Aspergillus versicolor* OUCMDZ-2738.

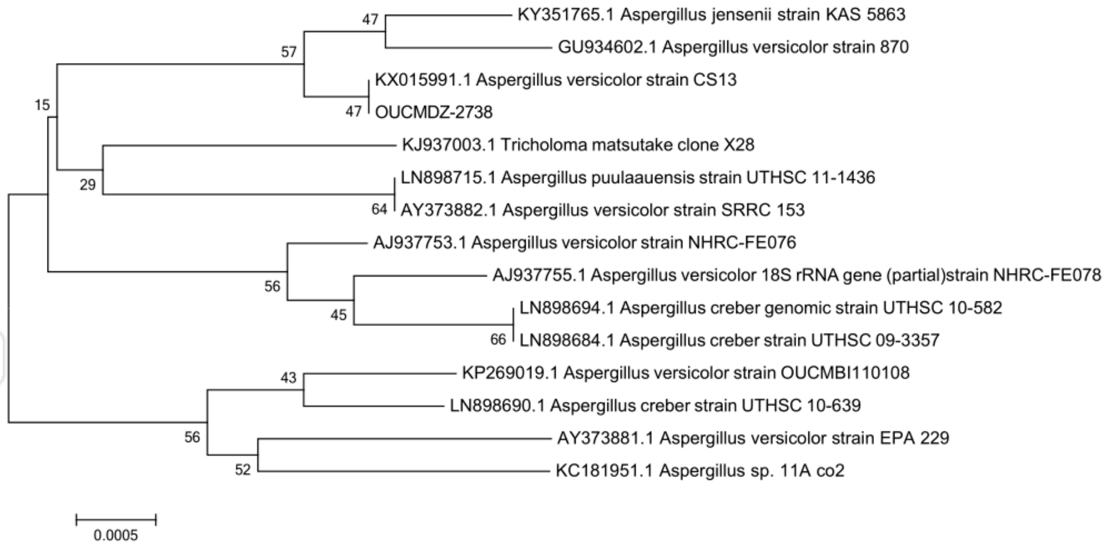

Figure S19. ^{1}H NMR spectrum of compound **12** in DMSO-d_6.

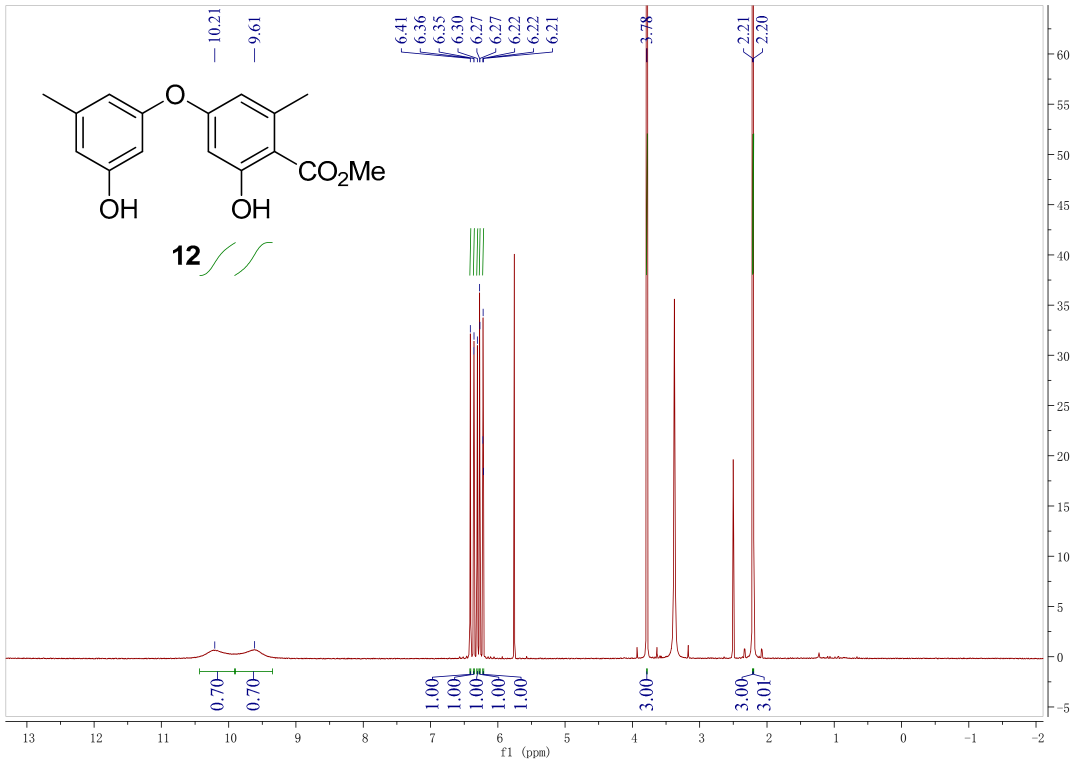

Figure S20. ^{13}C NMR spectrum of compound **12** in DMSO-d_6.

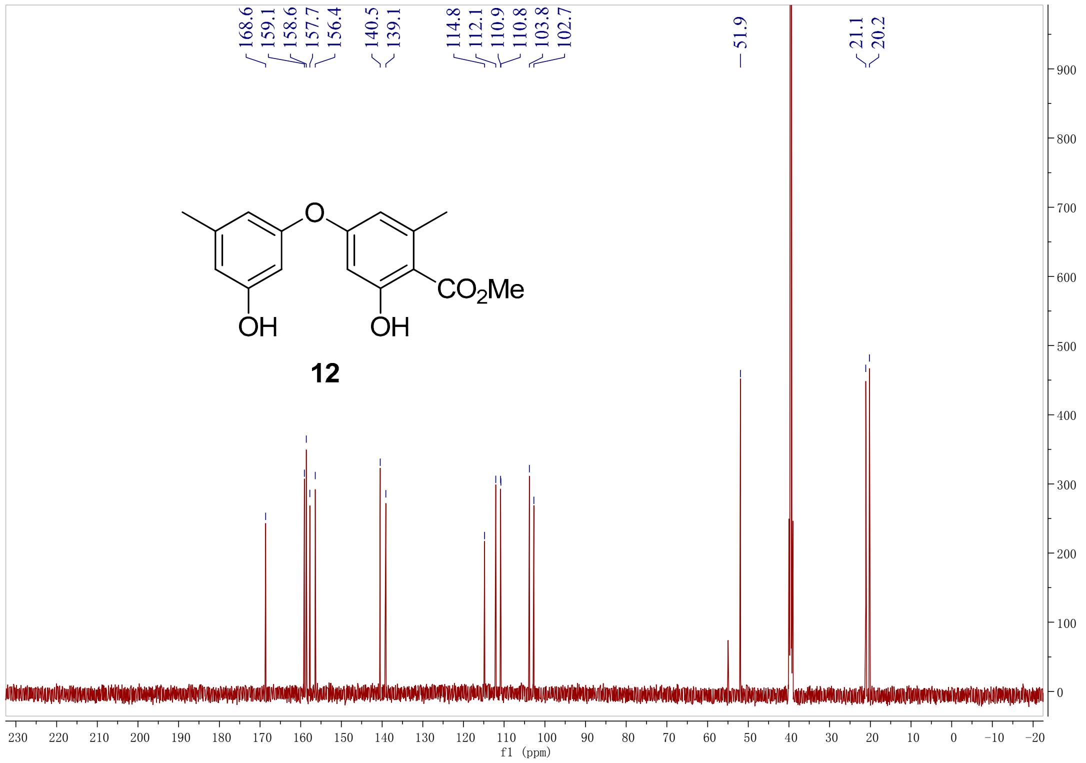

Figure S21. HSQC spectrum of compound **12** in DMSO-d_6.

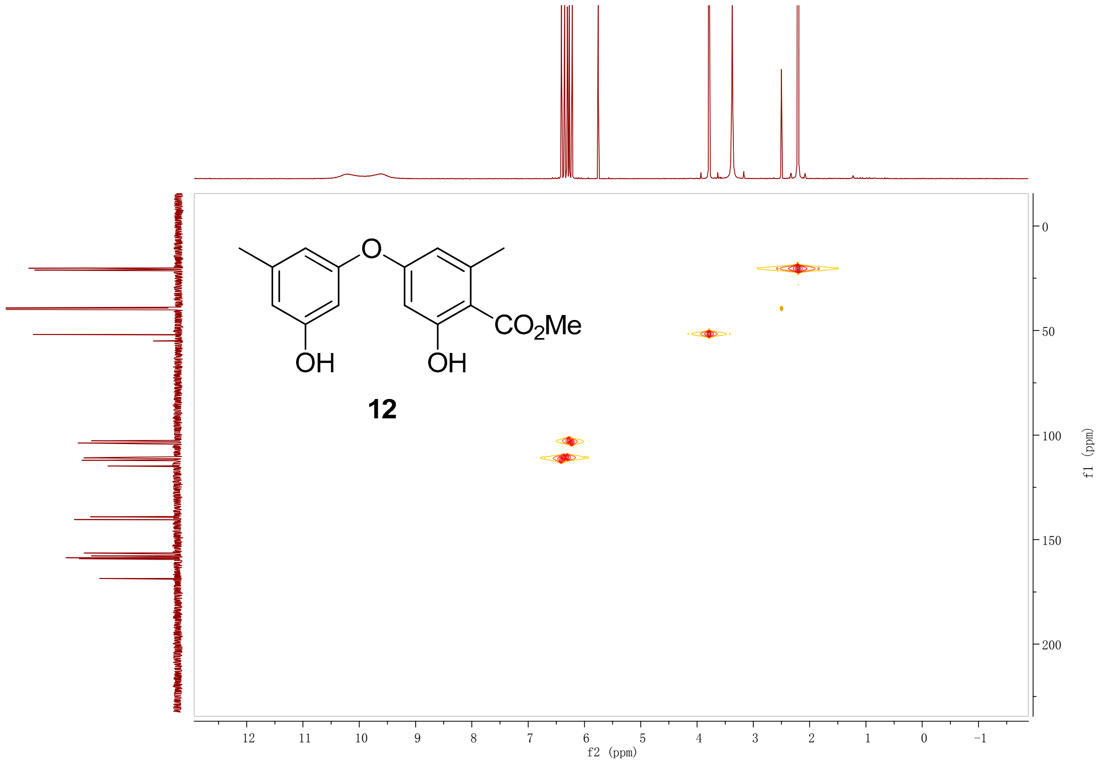

Figure S22. HMBC spectrum of compound **12** in DMSO-d_6.

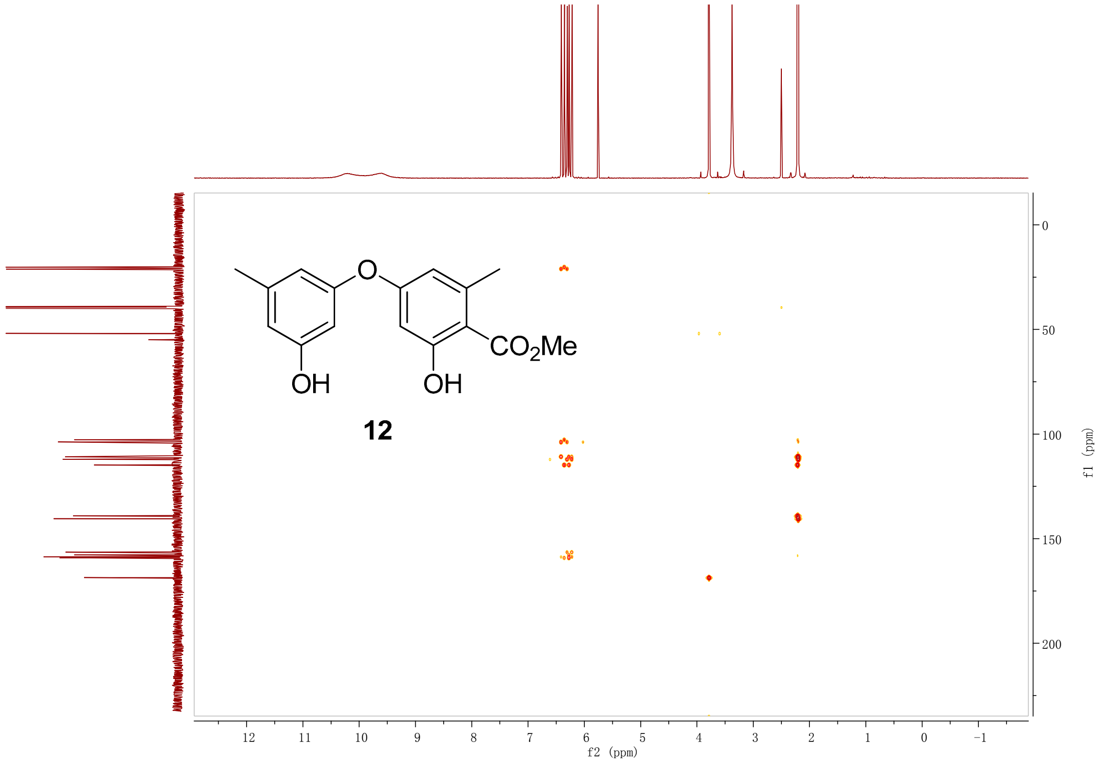

Figure S23. ROESY spectrum of compound **12** in DMSO-d_6.

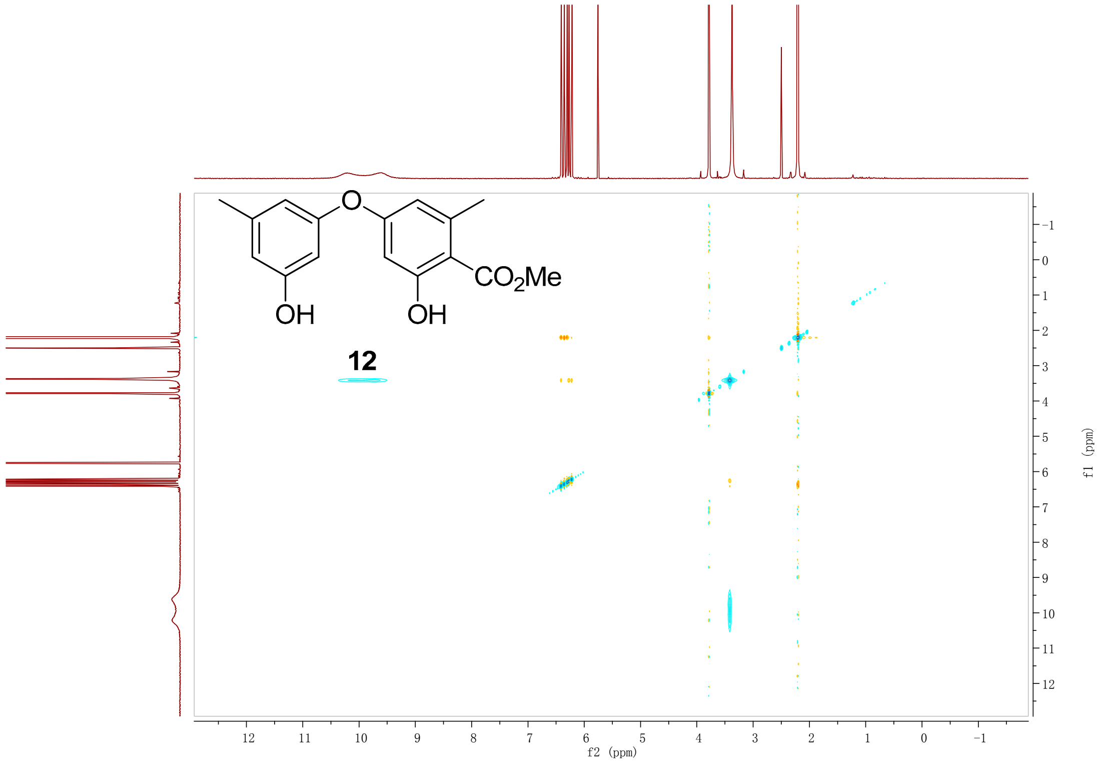

Figure S24. HRESIMS spectrum of compound **12a**.

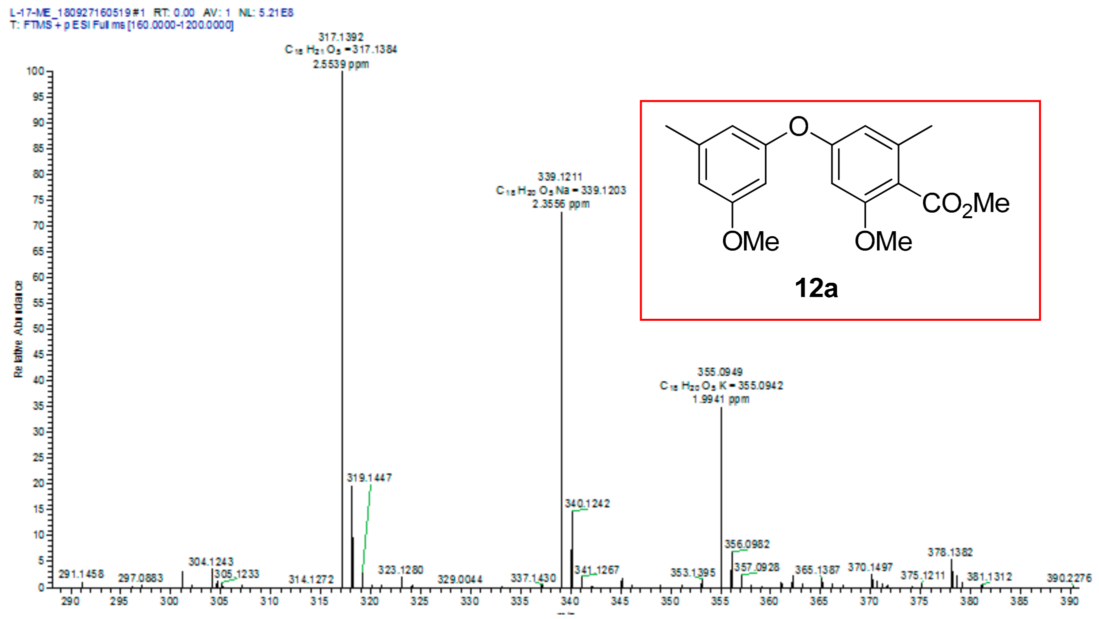

Figure S25. ^{1}H NMR spectrum of compound **12a** in DMSO-d_6.

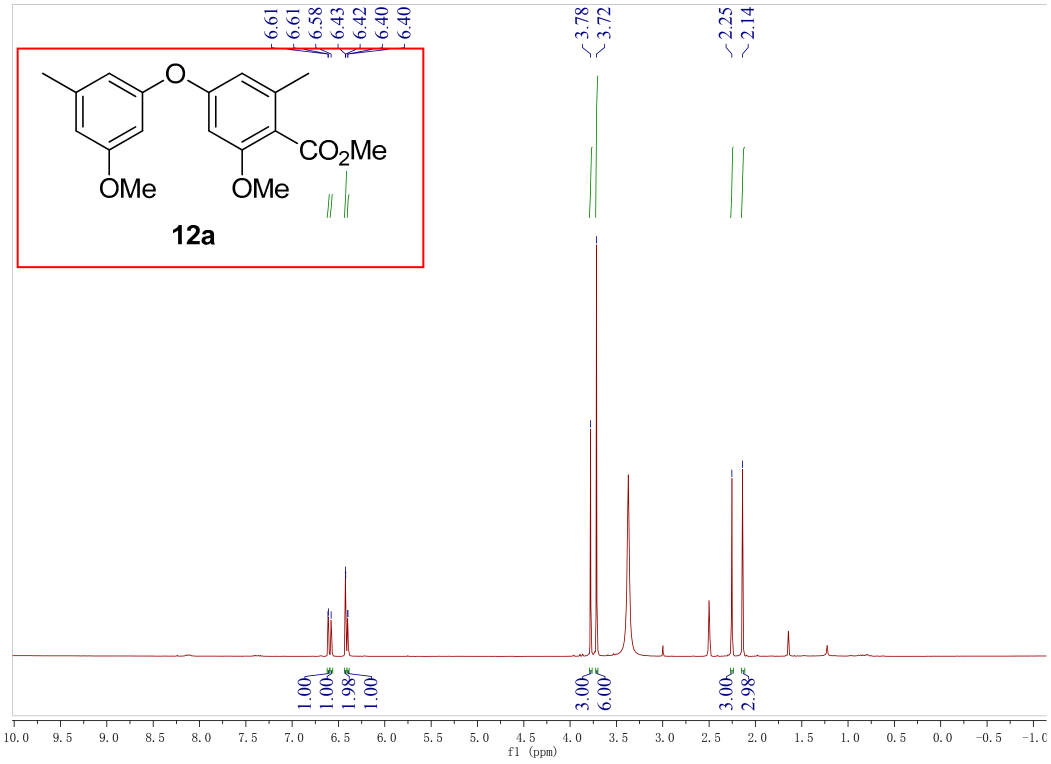

Figure S26. ^{13}C NMR spectrum of compound **12a** in DMSO-d_6.

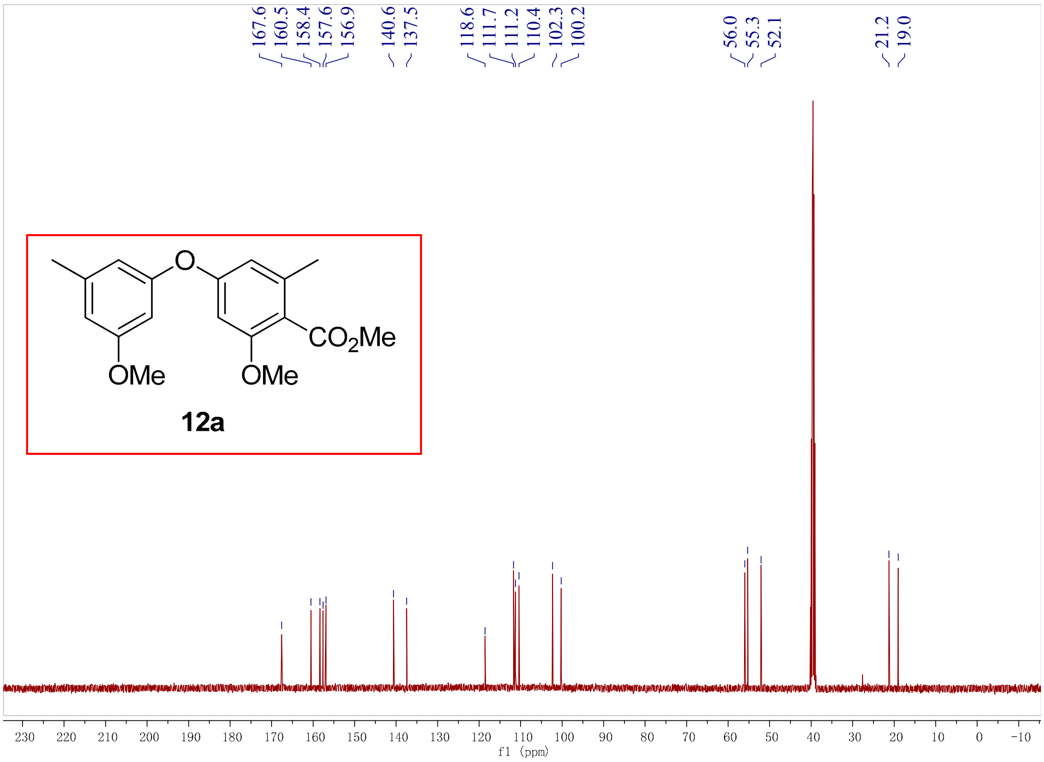

Figure S27. HSQC spectrum of compound **12a** in DMSO-d_6.

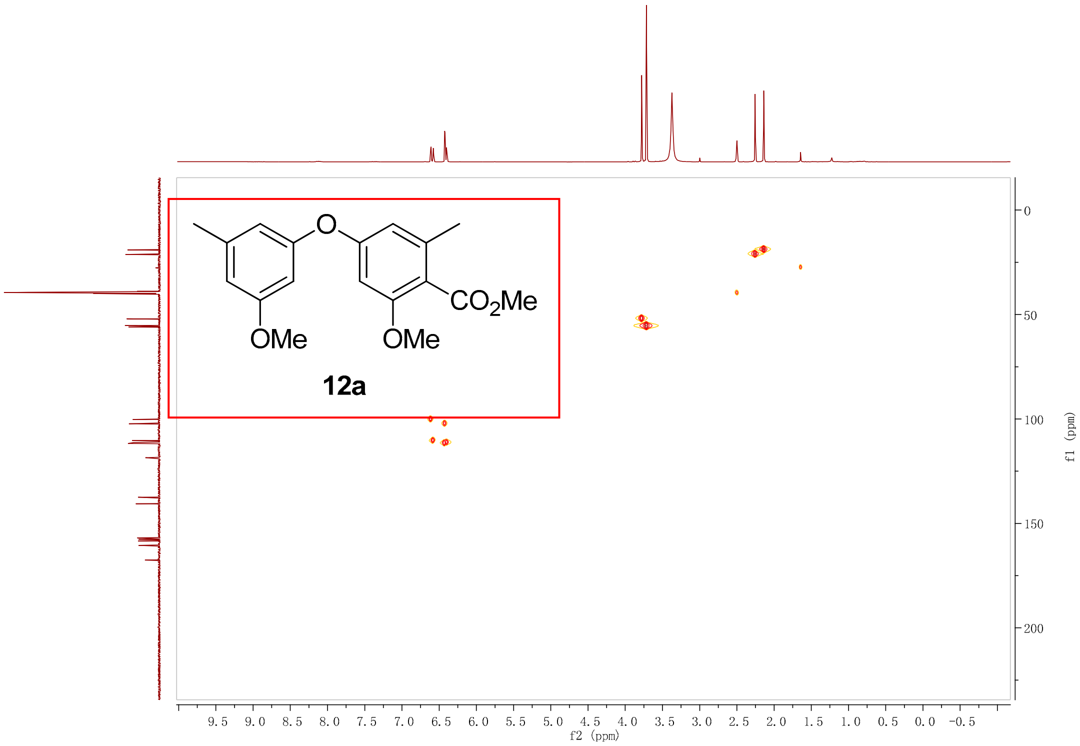

Figure S28. ROESY spectrum of compound **12a** in DMSO-d_6.

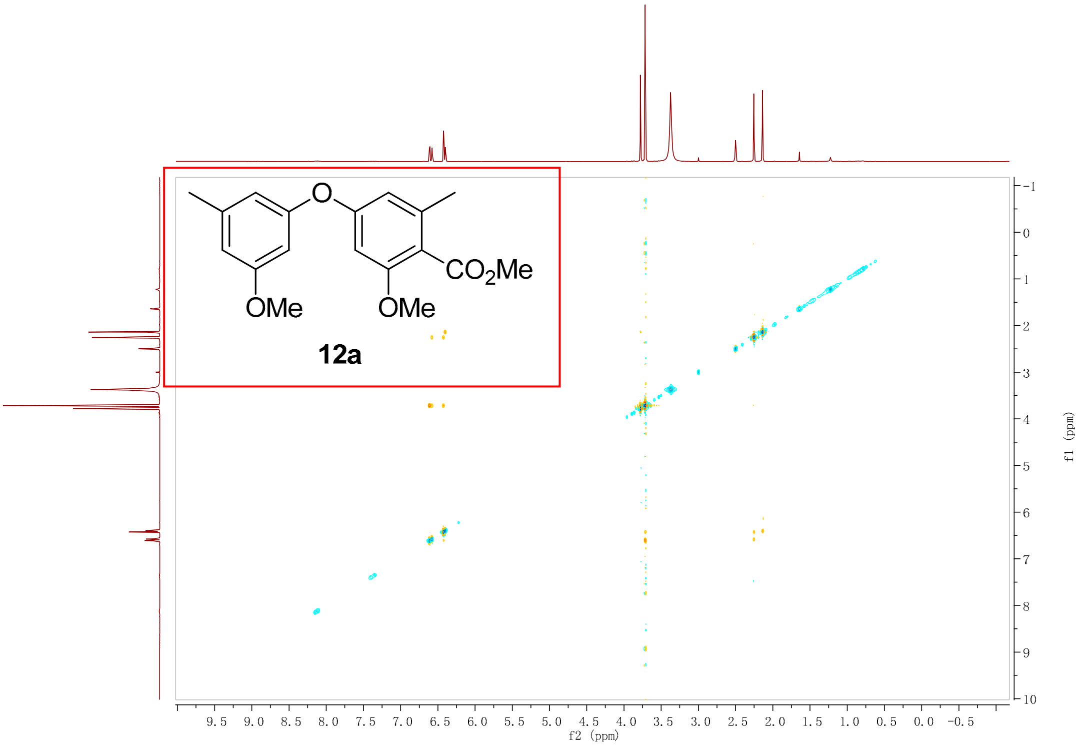

Figure S29. HMBC spectrum of compound **12a** in DMSO-d_6.

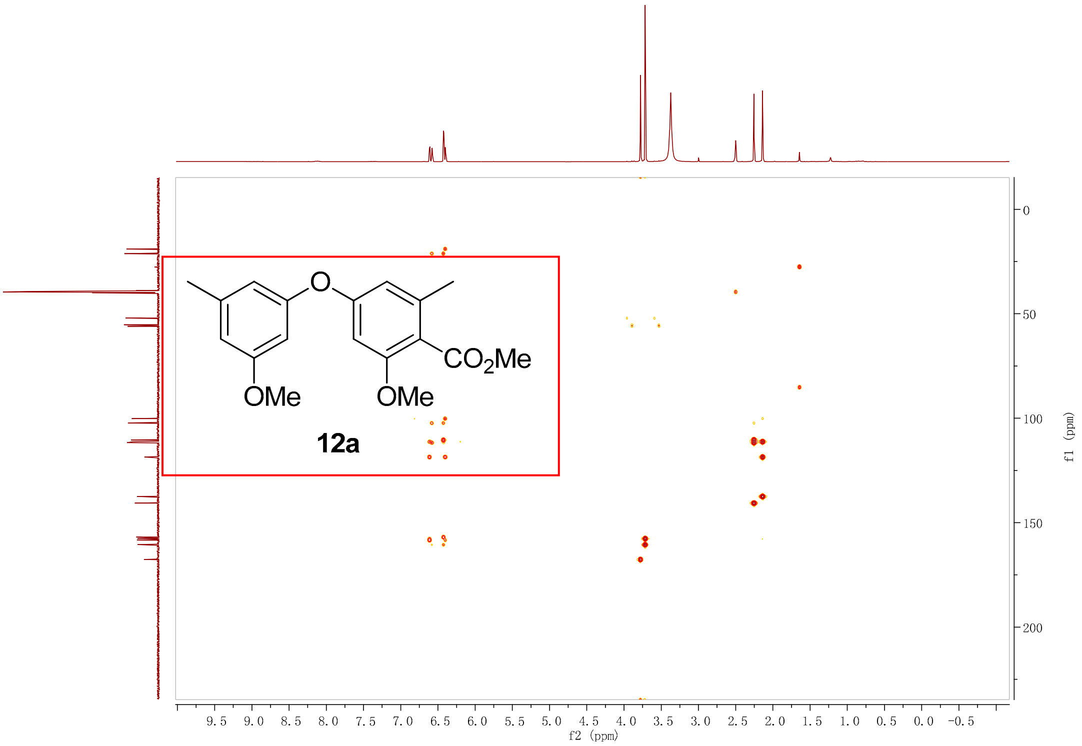

Figure S30. The UPLC–MS Analysis of Fermented Extracts.

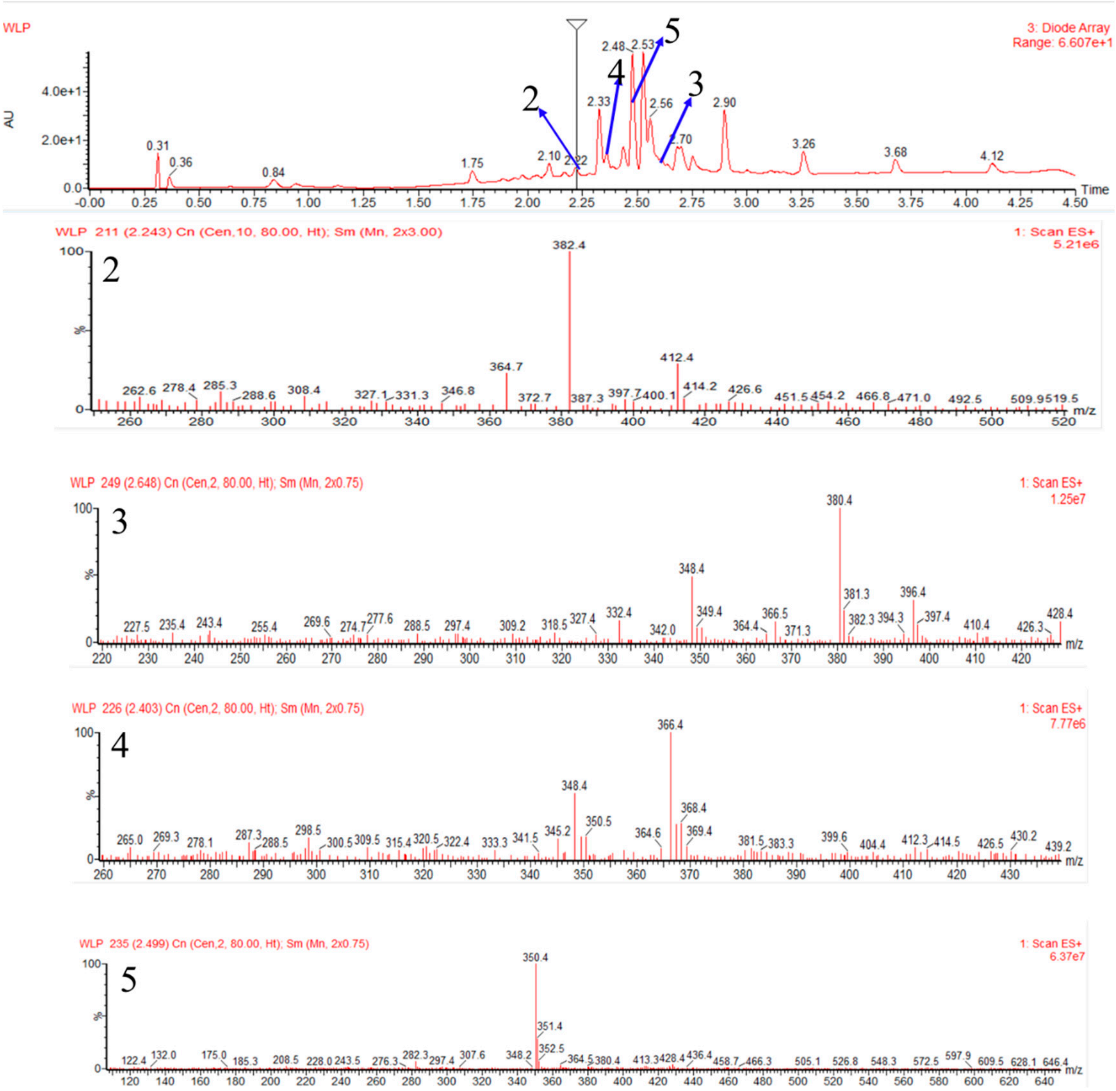

Figure S31. Retention times of compounds **1–12** in the same elution gradient with original extract fingerprint.

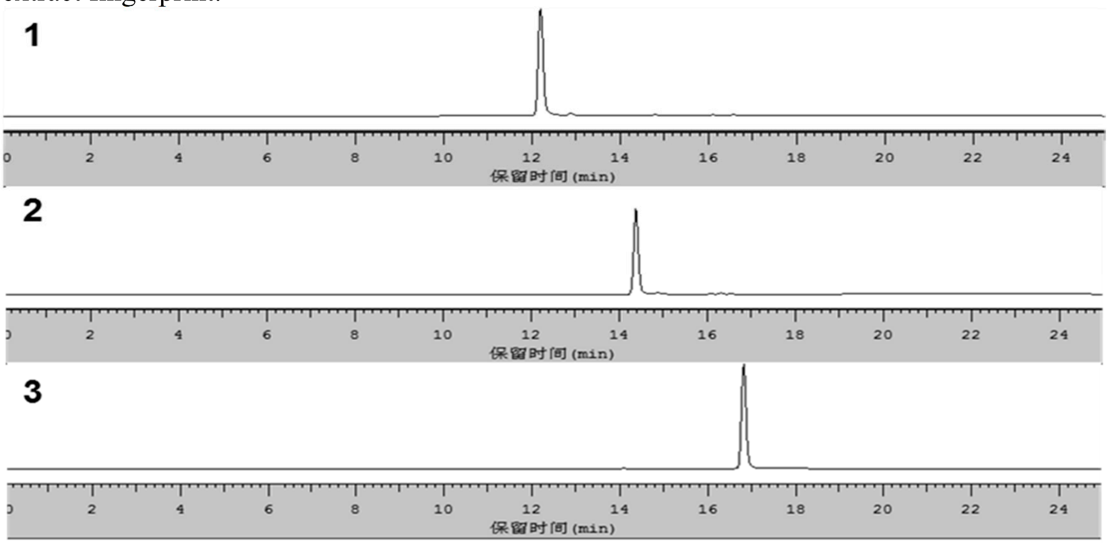

4

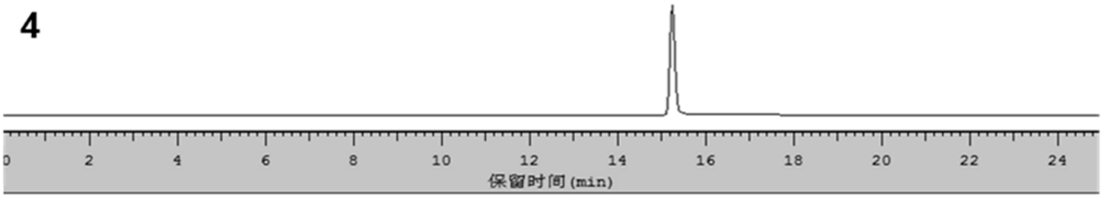

5

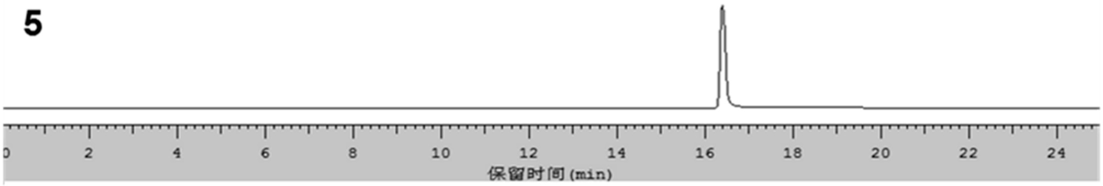

6

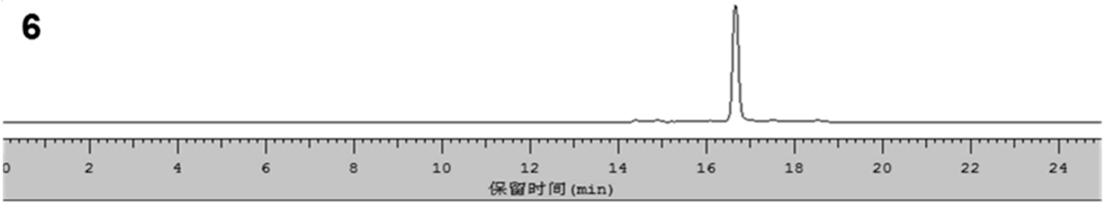

7

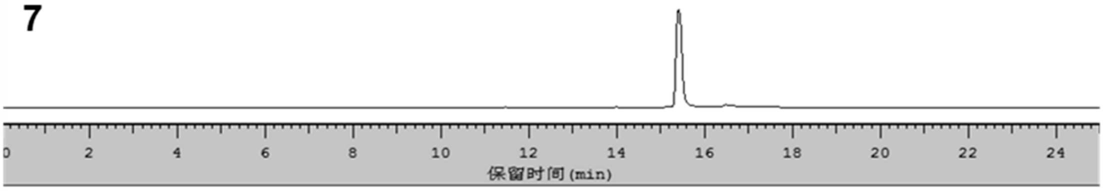

8

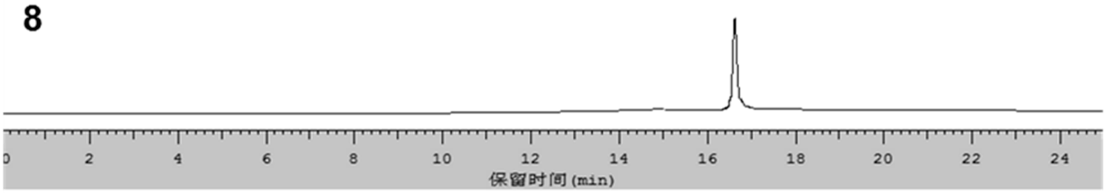

9

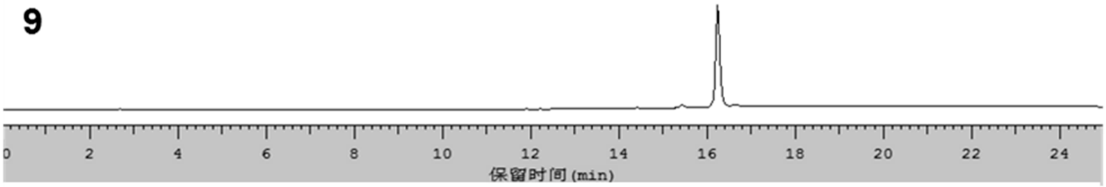

10

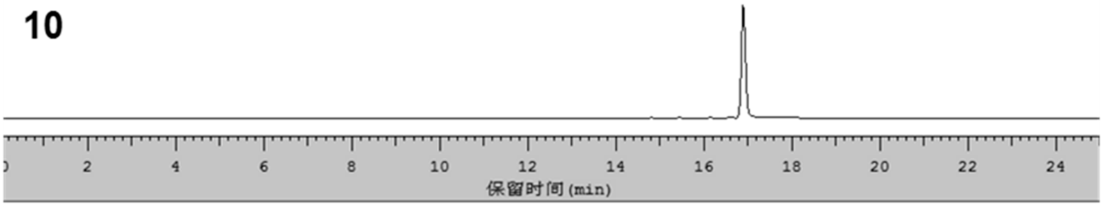

11

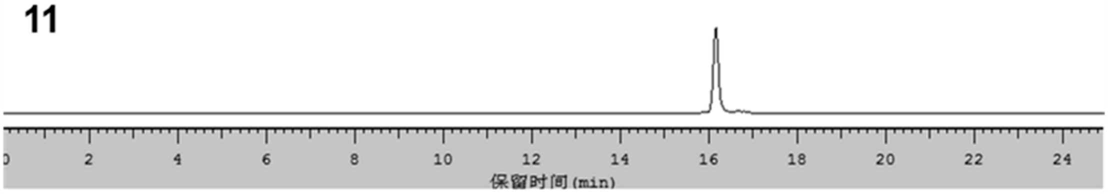

12

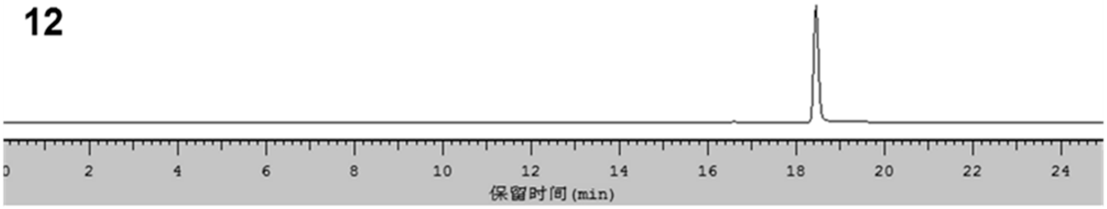

Conditions:

Column: YMC-Pack ODS-A column (S-5 μm, 12 nm, 250 × 4.6 mm, Shenzhen Chemist Technology Co. Ltd., Shenzhen, China).
Gradient: 5–100% MeOH in 0–15 min; 100% MeOH in 15–20 min; 0–95% H_2O in 20–25 min.
Column temperature: 30 °C.
Flow rate: 1 mL/min.
Detector: 254 nm
Injection volume: 5 μL

The physical properties of the known compounds (3–11):

Compound **3**: ^{1}H NMR (400 MHz, DMSO-d_6) δ 11.09 (s, 1H,18-NH), 9.45 (s, 1H, 2-NH), 7.42 (d, J = 7.9 Hz, 1H, H-16), 7.19 (d, J = 7.9 Hz, 1H, H-13), 7.09 (t, J = 7.9 Hz, 1H, H-15), 7.03 (t, J = 7.9 Hz, 1H, H-10), 7.00 (d, J = 7.3 Hz, 1H, H-14), 6.08 (dd, J = 17.1, 10.8 Hz, 1H, H-21), 5.07 (dd, J = 10.8, 1.1 Hz 1H, H_a-22), 5.03 (dd, J =17.1, 1.1 Hz 1H, H_b-22), 3.70 – 3.55 (m, 2H, H_2-6), 3.30 (s, 3H, CH_3O-9), 2.34 – 2.26 (m, 1H, H_a-8), 2.08 – 1.99 (m, 1H, H_b-8), 1.94 (dd, J = 15.6, 7.4 Hz, 2H, H_2-7), 1.49 (s, 3H, H_3-23), 1.45 (s, 3H, H_3-24). ^{13}C NMR (100 MHz, DMSO-d_6) δ 162.7 (C, C-1), 159.4 (C, C-4), 145.1 (C,C-19), 144.5 (CH, C-21), 135.1 (C, C-17), 126.2 (C, C-12), 125.2 (C, C-3), 120.8 (CH, C-15), 119.4 (CH, C-14), 119.0 (CH, C-13), 112.7 (CH, C-10), 111.7 (CH_2, C-22), 111.6 (CH, C-16), 103.8 (C, C-11), 91.4 (C,C-9), 51.3 (CH_3, CH_3O-9), 45.1 (CH_2, C-6), 39.0 (C, C-20), 32.4 (CH_2, C-8), 27.8 (CH_3, C-24), 27.4 (CH_3, C-23), 19.3 (CH_2, C-7).

Compound **4**: ^{1}H NMR (400 MHz, DMSO-d_6) δ 11.04 (s, 1H, 18-NH), 9.07 (s, 1H, 2-NH), 7.39 (d, J = 7.9 Hz, 1H, H-16), 7.34 (d, J = 7.9 Hz, 1H, H-13), 7.06 (t, J = 7.9 Hz, 1H, H-14), 6.98 (t, J = 7.9 Hz, 1H, H-15), 6.96 (s, 1H, H-10), 6.84 (s, 1H, 9-OH), 6.07 (dd, J = 17.1, 10.8 Hz, 1H, H-21), 5.06 (dd, J = 10.8, 1.1 Hz, 1H, H_a-22), 5.02 (dd, J = 17.1, 1.1 Hz, 1H, H_b-22), 3.68 – 3.59 (m, 1H, H_a-6), 3.50 (dt, J = 11.8, 8.3 Hz, 1H, H_b-6), 2.10 (dd, J = 9.6, 3.5 Hz, 2H, H_2-8), 2.03 (m, 1H, H_a-7), 1.90 (m, 1H, H_a-7), 1.49 (s, 3H, H_3-24), 1.45 (s, 3H, H_3-23).^{13}C NMR (100 MHz, DMSO-d_6) δ 165.3 (C, C-1), 159.4 (C, C-4), 145.2 (C, C-19), 144.3 (CH, C-21), 135.1 (C, C-17), 126.4 (CH, C-12), 125.7 (C, C-3), 120.7 (CH, C-15), 119.7 (CH, C-14), 119.3 (CH, C-13), 111.9 (CH, C-10), 111.7 (CH_2, C-22), 111.4 (CH, C-16), 104.1 (C, C-11), 86.6 (C, C-9), 44.7 (CH_2, C-6), 39.1 (C, C-20), 35.7 (CH_2, C-8), 27.8 (CH_3, C-24), 27.5 (CH_3, C-23), 19.5 (CH_2, C-7).

Compound **5**: ^{1}H NMR (400 MHz, DMSO-d_6) δ 11.04 (s, 1H, 18-NH), 8.94 (s, 1H, 2-NH), 7.43 (d, J = 7.9 Hz, 1H, H-16), 7.26 (d, J = 7.9 Hz, 1H, H-13), 7.09 (m, 1H, H-14), 7.01 (m, 1H, H-15), 6.93 (s, 1H, H-10), 6.09 (dd, J = 17.2, 10.7 Hz, 1H, H-21), 5.07 (dd, J = 10.7, 1.2 Hz, 1H, H_a-22), 5.04 (dd, J = 17.2, 1.2 Hz, 1H, H_b-22), 4.45 (m, 1H, H-9), 3.59 (m, 1H, H_a-6), 3.47 (dd, J = 11.7, 8.9 Hz, 1H, H_b-6), 2.21 (m, 1H, H_a-8), 1.93 (m, 1H, H_b-8), 1.88 (dd, J = 9.5, 4.9 Hz, 1H, H_a-7), 1.84 (m, 1H, H_b-7), 1.51 (s, 3H, H_3-23), 1.47 (s, 3H, H_3-24). ^{13}C NMR (100 MHz, DMSO-d_6) δ 166.0 (C, C-1), 158.4 (C, C-4), 145.2 (C, C-19), 144.1 (CH, C-21), 135.1 (C, C-17), 126.3 (C, C-12), 126.0 (C, C-3), 120.7 (CH, C-15), 119.3 (CH, C-14), 119.3 (CH, C-13), 111.6 (CH, C-10), 111.5 (CH_2, C-22), 110.6 (CH, C-16), 103.9 (C, C-11), 58.6 (CH, C-9), 44.9 (CH_2, C-6), 39.0 (C, C-20), 28.2 (CH_2, C-8), 27.7 (CH_3, C-24), 27.4 (CH_3, C-23), 21.6 (CH_2, C-7).

Compound **6**: yellow crystal; ^{1}H NMR (400 MHz, DMSO-d_6) δ 11.10 (s, 1H, 18-NH), 8.85 (s, 1H, 2-NH), 7.43 (d, J = 7.9 Hz, 1H, H-16), 7.20 (d, J = 7.9 Hz, 1H, H-13), 7.10 (t, J = 7.9 Hz, 1H, H-14), 7.01 (t, J = 7.9 Hz, 1H, H-15), 6.91 (s, 1H, H-10), 6.10 (d, J = 3.5 Hz, 1H, H-8), 6.07 (dd, J = 17.3, 10.6 Hz, 1H, H-21), 5.06 (dd, J = 10.6, 1.1 Hz, 1H, H_a-22), 5.03 (dd, J = 17.3, 1.1 Hz, 1H, H_b-22), 4.02 (t, J = 9.1 Hz, 2H, H_2-6), 2.77 (td, J = 9.5, 3.0 Hz, 2H, H_2-7), 1.47 (s, 6H, H_3-23, H_3-24). ^{13}C NMR (100 MHz, DMSO-d_6) δ 154.7 (C, C-1), 153.9 (C, C-3), 145.1 (C, C-19), 144.1 (CH, C-21), 135.1 (C, C-17), 133.7 (C, C-9), 126.0 (C, C-12), 125.8 (C, C-3), 120.8 (CH, C-15), 119.5 (CH, C-14), 119.0 (CH, C-13), 118.8 (CH, C-8), 111.7 (CH, C-10), 111.7 (CH_2, C-22), 110.2 (CH, C-16),

103.3 (C, C-11), 45.5 (CH_2, C-6), 39.0 (C, C-20), 27.7 (CH_2, C-7), 27.5 (CH_3, C-23), 27.5 (CH_3, C-24). ESI-MS *m*/*z* 370.2 $[M + Na]^+$.

Compound **7**: yellow solid, $[\alpha]_D^{25}$ –35.5 (*c* 4.84, MeOH); ^{1}H NMR (400 MHz, DMSO-d_6) δ 9.39 (s, 1H, 3-OH), 9.15 (s, 1H, 3′-OH), 6.37 (d, *J* = 2.4 Hz, 1H, H-4′), 6.29 (s, 1H, H-4), 6.19 (s, 1H, H-6), 6.11 (brs, 1H, H-2), 6.09 (d, *J* = 2.4 Hz, 1H, H-2′), 4.10 (s, 1H, 3′′-OH), 4.08 (d, *J* = 5.8 Hz, 1H, 2′′-OH), 3.36 (m, 1H, H-2′′), 2.64 (d, *J* = 12.4 Hz, 1H, H_a-1′′), 2.40 (dd, *J* = 13.5, 10.4 Hz, 1H, H_b-1′′), 2.27 (s, 3H, H_3-7′), 2.17 (s, 3H, H_3-7), 1.06 (s, 3H, H_3-5′′), 1.05 (s, 3H, H_3-4′′). ^{13}C NMR (100 MHz, DMSO-d_6) δ 158.5 (C, C-3), 158.4 (C, C-1), 155.6 (C, C-3′),155.2 (C, C-1′), 139.9 (C, C-5), 121.2 (C, C-6′), 112.7 (CH, C-4′), 110.5 (CH, C-4), 109.2 (CH, C-6),103.9 (CH, C-2′),102.1 (CH, C-2), 78.3 (CH, C-2′′), 72.0 (CH, C-3′′), 28.0 (CH_2, C-1′′), 25.6 (CH_3, C-4′′), 25.3 (CH_3, C-5′′), 21.3 (CH_3, C-7), 20.4 (CH_3, C-7′). ECD (0.0004 M, MeOH) λ_{max} ($\Delta\varepsilon$)220 (-4.9), 282 (-0.9) nm. ESI-MS *m*/*z* 331.1 $[M - H]^-$.

Compound **8**: yellow oil, $[\alpha]_D^{25}$ –24.1 (*c* 0.83, MeOH); ^{1}H NMR (400 MHz, DMSO-d_6) δ 9.35 (s, 1H, 3-OH), 9.35 (s, 1H, 3′-OH), 6.37 (d, *J* = 2.4 Hz, 1H, H-4′), 6.28 (s, 1H, H-4), 6.17 (s, 1H, H-6), 6.09 (brs, 1H, H-2), 6.07 (d, *J* = 2.4 Hz, 1H, H-2′), 4.29 (d, *J* = 5.5 Hz, 1H, 2′′-OH), 3.54 (dd, *J* = 8.1, 5.0 Hz, 1H, H-2′′), 3.07 (s, 3H, CH_3-3′′), 2.62 (d, *J* = 12.2 Hz, 1H, H_a-1′′), 2.38 (dd, *J* = 13.5, 10.4 Hz, 1H, H_b-1′′), 2.26 (d, *J* = 2.4 Hz, 3H, H_3-7), 2.16 (s, 3H, H_3-7′), 1.06 (s, 3H, H_3-5′′), 1.04 (s, 3H, H_3-4′′). ^{13}C NMR (100 MHz, DMSO-d_6) δ 158.5 (C, C-3), 158.5 (C, C-1), 155.7 (C, C-3′),155.2 (C, C-1′), 139.9 (C, C-5), 121.2 (C, C-6′), 112.7 (CH, C-4′), 110.5 (CH, C-4), 109.2 (CH, C-6),104.0 (CH, C-2′),102.1 (CH, C-2), 77.2 (CH, C-3′′), 75.6 (CH, C-2′′), 48.9 (CH_3, 3′-CH_3), 27.7 (CH_3, C-1′′), 21.5 (CH_3, C-7), 21.3 (CH_3, C-7′), 20.5 (CH_3, C-5′′), 20.4 (CH_3, C-4′′). ESI-MS *m*/*z* 345.2 $[M - H]^-$.

Compound **9**: yellow oil, $[\alpha]_D^{25}$ –5.3 (*c* 1.51, MeOH); ^{1}H NMR (400 MHz, DMSO-d_6) δ 9.35 (s, 1H, 3-OH), 9.35 (s, 1H, 3′-OH), 6.36 (d, *J* = 2.4 Hz, 1H, H-4′), 6.29 (s, 1H, H-4), 6.17 (s, 1H, H-6), 6.09 (brs, 1H, H-2), 6.08 (d, *J* = 2.4 Hz, 1H, H-2′), 4.72 (d, *J* = 3.8 Hz, 1H, 2′′-OH), 4.65 (d, *J* = 14.6 Hz, 2H, H_2-4′′), 4.05 (s, 1H, H-2′′), 2.63 (dd, *J* = 13.4, 5.6 Hz, 1H, H_a-1′′), 2.52 (m, 1H, H_b-1′′), 2.24 (d, *J* = 8.0 Hz, 3H, H_3-7′), 2.17 (s, 3H, H_3-7), 1.65 (s, 3H, H_3-5′′). ^{13}C NMR (100 MHz, DMSO-d_6) δ 158.5 (C, C-3), 158.4 (C, C-1), 155.8 (C, C-3′), 155.2 (C, C-1′), 148.6 (C, C-3′′), 139.9 (C, C-5), 139.7 (C, C-5′), 119.8 (C, C-6′), 112.7 (CH, C-4′), 110.5 (CH, C-4), 109.6 (CH_2, C-4′′), 109.0 (CH, C-6), 103.9 (CH, C-2′), 101.9 (CH, C-2), 74.8 (CH, C-2′′), 32.6 (CH_2, C-1′′), 21.2 (CH_3, C-7), 20.2 (CH_3, C-7′). ECD (0.0004 M, MeOH) λ_{max} ($\Delta\varepsilon$) 220 (-1.0), 282 (-0.3) nm.ESI-MS *m*/*z* 313.1 $[M - H]^-$.

Compound **10**: yellow oil; ^{1}H NMR (400 MHz, DMSO-d_6) δ 9.41 (s, 1H, 3-OH), 9.41 (s, 1H, 3′-OH), 6.41 (d, *J* = 2.3 Hz, 1H, H-4′), 6.29 (s, 1H, H-4), 6.11 (s, 1H, H-6), 6.10 (d, *J* = 2.3 Hz, 1H, H-2′), 6.05 (brs, 1H, H-2), 3.67 (s, 2H, H_2-1′′), 2.66 (m, 1H, H-3′′), 2.15 (s, 3H, H_3-7), 2.08 (s, 3H, H_3-7′), 0.97 (s, 3H, H_3-5′′), 0.95 (s, 3H, H_3-4′′). ^{13}C NMR (100 MHz, DMSO-d_6) δ 211.3 (C, C-2′′), 158.5 (C, C-3), 158.0 (C, C-1), 156.5 (C, C-3′), 155.1 (C, C-1′), 140.0 (C, C-5), 139.6 (C, C-5′), 116.1 (CH, C-4′), 112.6 (CH, C-4), 110.8 (C, C-6′), 109.1 (CH, C-6), 103.7 (CH, C-2′), 102.1 (CH, C-2), 39.4 (CH, C-3′′), 37.7 (CH_2, C-1′′), 21.2 (CH_3, C-7), 19.6 (CH_3, C-7′), 18.2 (CH_3, C-4′′), 18.2 (CH_3, C-5′′). ESI-MS *m*/*z* 313.2 $[M - H]^-$.

Compound **11**: yellow oil; ^{1}H NMR (400 MHz, DMSO-d_6) δ 9.45 (s, 2H, HO-3/3′), 6.33 (s, 2H, H-4/4′), 6.24 (s, 2H, H-6/6′), 6.15 (t, *J* = 2.0 Hz, 2H, H-2/2′), 2.18 (s, 6H, H_3-7/7′). ^{13}C NMR (100 MHz, DMSO-d_6) δ 158.5 (C, C-3/3′), 157.6 (C, C-1/1′), 140.1 (CH, C-5/5′), 111.2 (CH, C-4/4′), 110.1 (CH, C-6/6′), 103.0 (CH, C-2/2′), 21.2 (C, C-7/7′). ESI-MS *m*/*z* 229.2 $[M - H]^-$.
